# Supplementary material for: Expression of antenatal symptoms of common mental disorders in The Gambia and the UK: a cross-sectional comparison study
Source: BMJ Open. 2023 Jul 10;13(7):e066807. doi: 10.1136/bmjopen-2022-066807 (PMC10335499; doi:10.1136/bmjopen-2022-066807)
Supplement: Supplementary data [file bmjopen-2022-066807supp002.pdf]

Supplementary Material 2

Table sup 2 Antenatal Clinic Names, Type, Area and Language

Antenatal Clinic	Clinic Type	Area Type	Language
Banjuliding Health Centre	Minor Health Clinic	Urban	Mandinka
Essau Distract Hospital	District hospital	Urban	Wolof
Fajikunda Health Centre	Major Health Clinic	Urban	Wolof
Gunjur Health Centre	Minor Health Clinic	Rural	Mandinka
Kafuta Health Centre	Minor Health Clinic	Rural	Mandinka
Kuntair Health Centre	Minor Health Clinic	Rural	Wolof
Pirang Health Centre	Minor Health Clinic	Rural	Mandinka
Serekunda Health Centre	Major Health Clinic	Urban	Wolof
Sinchu Baliya Health Post	Community Clinic/Health Post	Urban	Wolof
Sukuta Health Centre	Minor Health Clinic	Urban	Mandinka

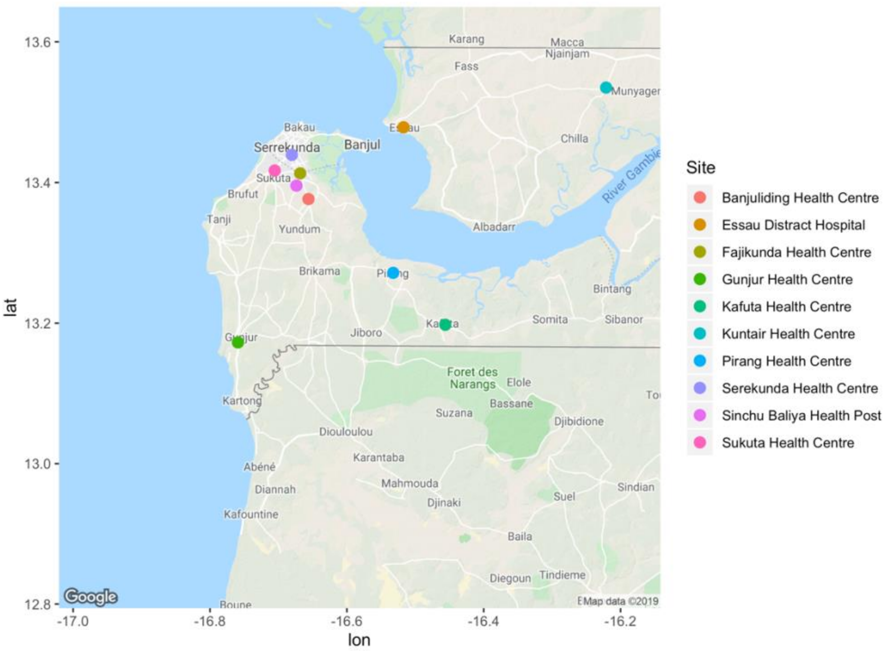

Fig sup 2. A map of the participating antenatal clinics described in Table sup 2.
