# Supplementary material for: Expression of antenatal symptoms of common mental disorders in The Gambia and the UK: a cross-sectional comparison study
Source: BMJ Open. 2023 Jul 10;13(7):e066807. doi: 10.1136/bmjopen-2022-066807 (PMC10335499; doi:10.1136/bmjopen-2022-066807)
Supplement: Supplementary data [file bmjopen-2022-066807supp003.pdf]

Supplementary Material 3

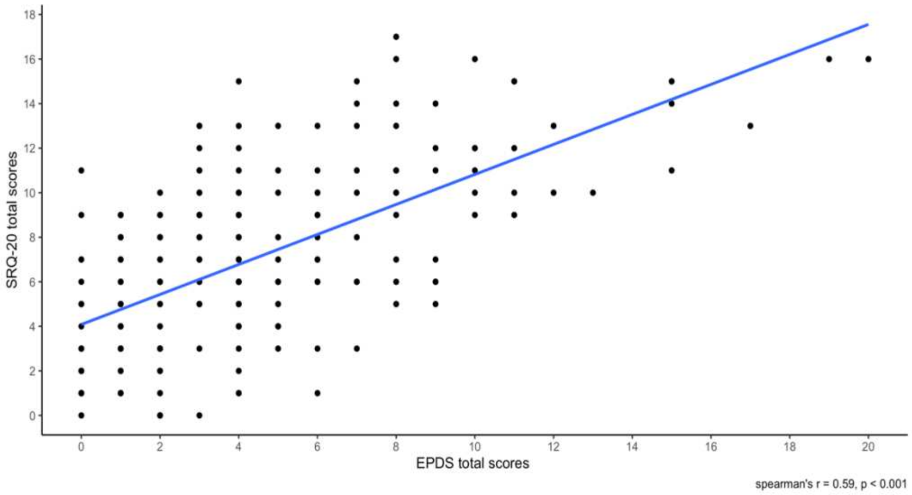

Fig Sup.3. Scatterplot of the correlation between Gambia EPDS and SRQ-20 total scores.
