# Supplementary material for: Expression of antenatal symptoms of common mental disorders in The Gambia and the UK: a cross-sectional comparison study
Source: BMJ Open. 2023 Jul 10;13(7):e066807. doi: 10.1136/bmjopen-2022-066807 (PMC10335499; doi:10.1136/bmjopen-2022-066807)
Supplement: Supplementary data [file bmjopen-2022-066807supp004.pdf]

Supplementary Materials 4

Table Sup.4.

Proportions of Specific Agreement and Kappa's

Cut-offs compared	Positive Agreement *n (%)*	Negative Agreement *n (%)*	Overall Agreement *n (%)*	Dis-agreement *n (%)*	Cohen's Kappa
EPDS ≥ 12 & SRQ20 ≥ 8	10 (19)	128 (76)	138 (60)	83 (36)	0.12
EPDS ≥ 12 & SRQ20 ≥ 7	10 (16)	107 (67)	117 (51)	104 (45)	0.09
EPDS ≥ 12 & SRQ20 ≥ 6	10 (13)	80 (55)	90 (39)	131 (57)	0.05
EPDS ≥ 10 & SRQ20 ≥ 8	21 (37)	128 (78)	149 (65)	72 (31)	0.25
EPDS ≥ 10 & SRQ20 ≥ 7	21 (31)	107 (70)	128 (56)	93 (41)	0.18
EPDS ≥ 10 & SRQ20 ≥ 6	21 (26)	80 (57)	101 (44)	120 (52)	0.11
EPDS ≥ 9 & SRQ20 ≥ 8	24 (40)	124 (77)	148 (65)	73 (32)	0.25
EPDS ≥ 9 & SRQ20 ≥ 7	25 (35)	104 (69)	129 (56)	92 (40)	0.19
EPDS ≥ 9 & SRQ20 ≥ 6	27 (32)	79 (58)	106 (46)	115 (50)	0.14
**Average Agreement**	**28%**	**67%**	**54%**	**43%**	

Note. Percent positive agreement = 2(number both ≥ cut off)/2(number both ≥ cut off) + total number of disagreement; Percent negative agreement = 2(number both ≤ cut off)/2(number both ≤ cut off) + total number of disagreement (Cicchetti & Feinstein, 1990)
