# Supplementary material for: Expression of antenatal symptoms of common mental disorders in The Gambia and the UK: a cross-sectional comparison study
Source: BMJ Open. 2023 Jul 10;13(7):e066807. doi: 10.1136/bmjopen-2022-066807 (PMC10335499; doi:10.1136/bmjopen-2022-066807)
Supplement: Author's manuscript [file bmjopen-2022-066807.draft_revisions.pdf]

BMJ Open

BMJ Open is committed to open peer review. As part of this commitment we make the peer review history of every article we publish publicly available.

When an article is published we post the peer reviewers' comments and the authors' responses online. We also post the versions of the paper that were used during peer review. These are the versions that the peer review comments apply to.

The versions of the paper that follow are the versions that were submitted during the peer review process. They are not the versions of record or the final published versions. They should not be cited or distributed as the published version of this manuscript.

BMJ Open is an open access journal and the full, final, typeset and author-corrected version of record of the manuscript is available on our site with no access controls, subscription charges or pay-per-view fees (http://bmjopen.bmj.com).

If you have any questions on BMJ Open's open peer review process please email info.bmjopen@bmj.com

Expression of Antenatal Symptoms of Common Mental Disorders in The Gambia and the UK

Journal:	*BMJ Open*
Manuscript ID	bmjopen-2022-066807
Article Type:	Original research
Date Submitted by the Author:	19-Jul-2022
Complete List of Authors:	Sanfilippo, Katie Rose; City University of London, Centre for Healthcare Innovation Research<br>Glover, Vivette; Imperial College London<br>Cornelius, Victoria; Imperial College London, Health and Social Care Research<br>Castro , Rita ; Universitat Zurich, Psychologisches Institut<br>McConnell, Bonnie ; Australian National University,<br>Darboe, Buba; Ministry of Health and Social Welfare The Gambia<br>Huma, Hajara; Ministry of Health and Social Welfare The Gambia; National Centre for Arts and Culture The Gambia<br>Ceesay, Hassoum ; National Centre for Arts and Culture<br>Ramchandani, Paul; University of Cambridge, Education<br>Cross, Ian; Cambridge University<br>Stewart, Lauren ; Goldsmiths University of London
Keywords:	MENTAL HEALTH, Depression & mood disorders < PSYCHIATRY, Anxiety disorders < PSYCHIATRY

SCHOLARONE™
Manuscripts

I, the Submitting Author has the right to grant and does grant on behalf of all authors of the Work (as defined in the below author licence), an exclusive licence and/or a non-exclusive licence for contributions from authors who are: i) UK Crown employees; ii) where BMJ has agreed a CC-BY licence shall apply, and/or iii) in accordance with the terms applicable for US Federal Government officers or employees acting as part of their official duties; on a worldwide, perpetual, irrevocable, royalty-free basis to BMJ Publishing Group Ltd ("BMJ") its licensees and where the relevant Journal is co-owned by BMJ to the co-owners of the Journal, to publish the Work in this journal and any other BMJ products and to exploit all rights, as set out in our licence.

The Submitting Author accepts and understands that any supply made under these terms is made by BMJ to the Submitting Author unless you are acting as an employee on behalf of your employer or a postgraduate student of an affiliated institution which is paying any applicable article publishing charge ("APC") for Open Access articles. Where the Submitting Author wishes to make the Work available on an Open Access basis (and intends to pay the relevant APC), the terms of reuse of such Open Access shall be governed by a Creative Commons licence – details of these licences and which Creative Commons licence will apply to this Work are set out in our licence referred to above.

Other than as permitted in any relevant BMJ Author's Self Archiving Policies, I confirm this Work has not been accepted for publication elsewhere, is not being considered for publication elsewhere and does not duplicate material already published. I confirm all authors consent to publication of this Work and authorise the granting of this licence.

For peer review only

Expression of Antenatal Symptoms of Common Mental Disorders in The Gambia and the UK

Katie Rose M. Sanfilippo[1], Vivette Glover[2], Victoria Cornelius[3], Rita Amiel Castro[4], Bonnie McConnell[5,] Buba Darboe[6], Hajara B. Huma[6,7], Hassoum Ceesay[7], Paul Ramchandani[8] , Ian Cross[9], Lauren Stewart[10]

[1]*Centre for Healthcare Innovation Research, City, University of London, UK*

[2]*Institute of Reproductive and Developmental Biology, Imperial College London, UK*

[3]*Imperial Clinical Trails Unit, School of Public Health, Imperial College London, UK*

[4] *Department of Clinical Psychology and Psychotherapy, Institute of Psychology, University of Zurich, Switzerland*

[5]*School of Music, Australian National University, Canberra, New South Wales, Australia*

[6]*The Ministry of Health and Social Welfare, The Gambia*

[7]*The National Centre for Arts and Culture, The Gambia*

[8]*Faculty of Education, University of Cambridge, UK*

[9]*Faculty of Music, University of Cambridge, UK*

[10]*Psychology Department, Goldsmiths, University of London, UK*

Correspondence concerning this paper should be addressed to Katie Rose M. Sanfilippo City, University of London at katie-rose.sanfilippo@city.ac.uk

Word Count: 3,699

For peer review only - http://bmjopen.bmj.com/site/about/guidelines.xhtml

ABSTRACT

Objectives

It is important to be able to detect symptoms of common mental disorders (CMDs) in pregnant women. However, the expression of these disorders can differ across cultures and depend on the specific scale used. This study aimed to (a) compare Gambian pregnant women's responses to the Edinburgh Postnatal Depression Scale (EPDS) and Self-reporting Questionnaire (SRQ-20) and (b) compare responses to the EPDS in pregnant women in The Gambia and UK.

Design

This comparative study investigates differences and similarities between Gambian EPDS and SRQ-20 scores by examining score distributions and the proportion of women with high levels of symptoms. Comparisons between the UK and Gambian EPDS scores were made through descriptive item analysis and exploratory factor analyses.

Setting

This study took place in The Gambia, West Africa and London, UK.

Participants

221 pregnant women from The Gambia completed both the SRQ-20 and the EPDS. 368 pregnant women from the UK completed the EPDS.

Results

Gambian participants' EPDS and SRQ-20 scores had different distributions and proportions of women identified as of concern (SRQ-20 = 42% versus EPDS = 5% using highest cut-off score). UK participants had higher EPDS scores than the Gambian participants ($p < 0.001$, 95%CIs [1.54, 2.84], $d = 0.53$) and a different factorial structure.

Conclusions

For peer review only - http://bmjopen.bmj.com/site/about/guidelines.xhtml

The differences in responses from Gambian pregnant women to the EPDS and SRQ-20 and the different EPDS responses between pregnant women in the UK and The Gambia further emphasises how methods and understanding around measuring perinatal mental health symptoms developed in Western countries need to be applied with care in other cultures

Keywords:

Perinatal; Common Mental Disorders; EPDS; SRQ-20; The Gambia; United Kingdom

ARTICLE SUMMARY

Strengths and limitations of this study

- Findings support studies conducted in other parts of Africa, adding to the literature by exploring differences within a new cultural context: The Gambia.
- This study compares two different scales (EPDS and SRQ-20) within the same cultural context in addition to comparing one scale (EPDS) across cultures.
- This study includes a fairly large sample size across The Gambia collected across 10 antenatal clinics across the West Coast and North Bank regions.
- EPDS and SRQ-20 have not been validated against a clinical gold-standard (clinical interview) to screen for possible CMD.

BACKGROUND

It is important to be able to recognise and treat symptoms of common mental disorders (CMDs), including depression and anxiety, during pregnancy. These symptoms affect not only the mother but can also have long-term adverse effects on her child [1]. Perinatal mental health problems are a particular challenge in low- and middle-income countries (LMICs) where they can be at least

For peer review only - http://bmjopen.bmj.com/site/about/guidelines.xhtml

twice as frequent as in higher-income countries and are often associated with stigma [2]. Thus, it is necessary to be able to identify these disorders both for intervention and research purposes. Short, simple self-rating questionnaires are often the easiest way to do this. However, women in different cultures and settings can have a different experience and presentation of CMD symptoms which may lead to under recognition or misidentification of psychological distress [3], impacting where women might seek support and what treatment options they are offered, if any. For instance, Oates et al [4], in a qualitative cross-cultural study of postnatal depression, found that while all cultures described a condition of 'morbid unhappiness' after childbirth, not everyone saw it as an illness remediable by health interventions. Likewise, the presentation of symptoms is not uniform across different populations. Previous work in different African populations have suggested that CMD symptoms are experienced in a more somatic or bodily way [5].

The EPDS, a commonly used tool to measure CMD symptoms, deliberately minimizes items of a somatic nature since it has been argued that physical changes in pregnancy might confound interpretation of CMD symptoms [6]. Even so, it has been recommended specifically for perinatal depression detection in LMICs [7]. However, Cox, Holden and Henshaw [6] note that the use of the EPDS in certain cultures may be inappropriate. Given that some populations experience CMD symptoms in a more bodily way, the use of a scale which precludes their detection is likely to underestimate the incidence of CMD symptoms in such populations. An alternative to the EPDS is the Self Reporting Questionnaire (SRQ-20), developed by the World Health Organisation for the detection of symptoms of common mental disorders in LMICs [8], and is suggested to be useful for measuring perinatal CMDs in Africa due to its inclusion of somatic symptom items [9,10].

For peer review only - http://bmjopen.bmj.com/site/about/guidelines.xhtml

While the somatization of CMDs has been documented in a number of African populations [5,9,11], the existing bias for research studies to concentrate on western populations, as well as the diversity of the African continent, motivates exploring this question in different populations and with different methodological approaches. In the current study we compare how pregnant women of Gambian nationality (and living in the Gambia) scored on two common measures of CMD symptoms, the EPDS and the SRQ-20. We also compare CMD symptoms, as measured by the EPDS, of these Gambian women against those of UK nationals (living in London, UK). Therefore, the objectives of this study were to explore differences between two scales within the same cultural context (The Gambia) and one scale across two different contexts (The UK and The Gambia).

METHODS

Participants

A convenience sample of 221 Gambian pregnant women between 10-30 weeks gestation (145 = Mandinka speaking and 76 = Wolof speaking) were assessed from May – August 2018. The Gambia is country on the West African coast and is made up of eight different ethnic groups all with their own languages and culture. Islam is the predominant religion in The Gambia and is a powerful factor in shaping lifeways, beliefs, and expectations. Participants were recruited from 10 different antenatal clinics across the western part of The Gambia (for more information see Supplementary Material 1). Any woman who attended the clinic, was above 18 years old and spoke either Mandinka or Wolof was approached. Women were excluded if they had previous or current psychosis or a history of a late term miscarriage.

A convenience sample of 368 pregnant women living in the UK were recruited from Queen Charlotte's and Chelsea Hospital in London as part of a previous longitudinal study. Exclusion

For peer review only - http://bmjopen.bmj.com/site/about/guidelines.xhtml

criteria included not speaking and/or writing English, not having access to a device with internet access, multiple pregnancy, in vitro fertilization, severe medical problems, pregnancy medical problems (including abnormal foetus), and severe psychiatric problems (e.g. psychosis, suicidality or drug addiction).

Measurement scales

The EPDS [12] is a ten-item scale that was designed to measure postnatal depression and anxiety symptoms that has subsequently been validated for antenatal use [13]. It has been used with perinatal populations across Africa [14] and in perinatal populations in The Gambia [15–18], though a validated version in Mandinka or Wolof could not be obtained. Participants answer on a four-point Likert-scale (from 0 – 3) how often they have experienced a specific symptom within the last week. The three anxiety items (items 1, 2, and 4) have been shown to form a valid anxiety subscale (EPDS-3 [19]). A higher total score (out of a total of 30) corresponds to a higher level of symptoms.

The SRQ-20 [8] is a 20-item scale which measures symptoms of CMDs. It was developed to be used in primary care settings and in LMICs and has also been used in perinatal populations across Africa [9,10,20] and The Gambia [17], though, in The Gambia, it has not been validated against a gold-standard. The SRQ-20 includes items measuring common somatic symptoms associated with anxiety and depression (headaches, low appetite, poor digestion, and sleep problems) as well as other psychological and physical symptoms (feeling frightened, unhappy, worthless, and low-energy) [8,9]. To each item, participants answer either yes = 1 or no = 0. The scores range from 0 – 20 with a higher score indicating higher levels of CMD symptoms.

For peer review only - http://bmjopen.bmj.com/site/about/guidelines.xhtml

Both scales were translated into Mandinka and Wolof using methods based on recommendations from the WHO [21], Hanlon et al., [10], and Cox, Holden and Henshaw [6]. First, the scales were translated into Mandinka and Wolof by bilingual experts. The terms used and concepts around mental distress were informed by a large qualitative study held prior to this work. An expert panel, who are experts in Gambian culture and language and a partner from the Ministry of Health and Social Welfare who is an expert in mental health, held a discussion and then refined the translation. Both were then back-translated into English and reviewed again by the expert panel that developed the final version found in Supplementary Material 2.

Procedure

Local midwives in The Gambia identified possible participants. If participants met the inclusion criteria, they were given information about the study by the two research assistants (RAs). Both RAs were trained psychiatric nurses and one was also a midwife. Verbal informed consent was obtained, witnessed, and recorded via RA signature or participant thumbprint. Due to low levels of literacy in The Gambia [22], participants' EPDS and SRQ-20 responses were collected orally by the RAs in alternating order per participant. For the UK sample, eligible participants were invited to take part by the researcher and written informed consent obtained. EPDS scores were measured online at about 21 weeks gestation. See Sanfilippo et al., [23] for more details about how disclosure and other ethical considerations were managed in The Gambia.

Statistical analysis

All statistical analysis was run using R [24]. To investigate differences in the EPDS and the SRQ-20 within the Gambian sample, differences in the distributional properties were examined

For peer review only - http://bmjopen.bmj.com/site/about/guidelines.xhtml

and the average score of the EPDS-3 subscale, and its contribution to the total EPDS score of the sample, was computed. A Bland Altman analysis was conducted to look at the agreement between the two scales [25]. To explore the impact of the somatic SRQ-20 items, the average score of the combined somatic items and their contribution to the total SRQ-20 score was calculated. Previous research has validated optimum cut-off scores for identifying perinatal CMDs within different populations in Africa [7,14]. The scores of 6, 7 and 8 were chosen as cut-offs for the SRQ-20, based on previous validation research in Ethiopia and Malawi [9,10]. Proportions of specific agreement and kappa statistics based on the identification of participants below or above these different cut offs were calculated based on methods explained by Cicchetti and Feinstein [26]. Kappas were calculated.

To investigate differences across cultures using the EPDS, differences in the distributional properties were explored. Three cut-off scores, 9, 10 and 12, were chosen for the EPDS based on previous research in the UK [6] and Africa [14].The average score of the EPDS-3 subscale was calculated, and its contribution to the total EPDS score of the sample was computed. Two exploratory factor analyses (EFA), one for each sample's EPDS scores, were performed to investigate the underlying pattern of the relationships between the ten observed items within the EPDS for the UK and Gambian samples [27]. A principal axis factoring extraction method was used as this method accounts for skewed data and is not bound to any distributional assumptions [27]. Polychoric correlations were used to account for the ordinal nature of the data, and an oblique rotation was chosen as correlations between factors were likely to exist [27].

Patient and Public Involvement

For peer review only

For peer review only - http://bmjopen.bmj.com/site/about/guidelines.xhtml

We engaged with patients and the public and relevant stakeholders in The Gambia to inform the research approach. This included how to best administer the questionnaires, the best way to recruit participants and potential cultural considerations which need to be made. To do this we held a half day meeting in the Gambia with delegates from the Ministry of Health and Social Welfare, the National Centre for Arts and Culture, a local obstetrician, patient advocate groups and students in Public Health and Psychiatric Nursing.

RESULTS

Demographic Information

Table 1 shows the demographic characteristics of the Gambian and UK samples. UK participants were significantly older ($p < 0.001$, 95%CIs [5.40, 7.21], $d = 1.35$) and had fewer children ($p < 0.001$, 95%CIs [0.64, 1.22], $d = 0.61$) and pregnancies ($p < 0.001$, 95%CIs [2.03, 3.59], $d = 0.86$) than the Gambian participants.

Table 1. Demographic Information for Gambian and UK Samples

	**Gambian Sample (n=221)** ***M (SD)***	**UK Sample (n=314)** ***M (SD)***
**Age***	27.27 (5.87)	33.4 (4.34)
**GA**	22.91 (5.45	21.07 (1.35)
**Parity***	2.34 (2.15)	1.48 (0.73)
**Gravida***	3.49 (2.25)	2.03 (2.03)

**Gambian Sample**	**Total** ***n (% of 221)***
**Marital Status**	
Single/Divorced/Widowed	6 (3%)
Married (Monogamous)	160 (72%)
Married (Polygamous)	55 (25%)
**Education Level**	

For peer review only

None	13(6%)
Informal (Arabic)	101 (46%)
Primary	29 (13%)
Secondary/Tertiary	78 (35%)
**Occupation**	
Housewife	138 (62%)
Other	82 (37%)
**Husband's Occupation**	
Skilled work	97(44%)
Manual/Trade Work	122 (55%)
**UK Sample**	***n(% out of 368)***
**Marital Status**	
Married/Civil Partnership	233 (74%)
Living with Partner	64 (20%)
Single	11 (4%)
Other	6 (2%)
**Education Level**	
Left before GCSE/O-Levels	2 (1%)
GCSE/O-Levels	16 (5%)
A-Levels	23 (7%)
Vocational Training	13 (4%)
University Degree	159 (51%)
Higher Degree	102 (32%)
**Occupation**	
Professional	197 (63%)
Managerial	46 (15%)
Skilled Non-Manual	16 (5%)
Skilled Manual	13 (4%)
Partly Skilled	10 (3%)
Not Applicable	22 (7%)
Other	10 (3%)
**Ethnic Group**	
Caucasian	209 (67%)
Indian/Pakistani/Bangladeshi	21 (7%)
Middle Eastern	12 (4%)
Afro/Afro-Caribbean	12 (4%)
South American/Hispanic	4 (1%)
Far Eastern	3 (1%)
Mixed	12 (4%)
Other	41 (13%)

For peer review only

For peer review only - http://bmjopen.bmj.com/site/about/guidelines.xhtml

Note. GA = Gestational Age. Primip = Primiparious. Housewife includes women who stated they did not have a job ("None"). All categories were informed by the categorizations used by the Ministry of Health and Social Welfare (MoHSW) in The Gambia. GCSE = General Certificate of Secondary Education. UK participants with missing demographic data (*n* = 54) were taken out for this summary. *Indicates a significant difference ($p<0.05$) between the groups

Comparing the EPDS and the SRQ-20 in The Gambian Sample

Figure 1 compares the total scores on the EPDS and the SRQ-20 from the Gambian sample.

Figure 1. Histogram of different cut-off scores for EPDS and SRQ-20.

A Bland and Altman analysis found low agreement between the EPDS and the SRQ-20 (lower limit of agreement = -3.80, CIs[-3.04, -4.54] and upper limit of agreement = 9.11, CIs [9.86, 8.37]). (See Supplementary Material 3). There was also low agreement between the two scales in the Gambian sample in identifying a case using the various cut-offs (kappa's ranging from 0.05-0.25) (See Supplementary Materials 4). The average positive agreement – when both scales detected a participant above the cut-off – was low (28%). Negative agreement – when both identified the participant as below the cut-off – was higher (67%). The average overall agreement – positive and negative agreement combined – was 54%. Supplementary Material 4 displays the proportion of participants identified using different cut-off scores, proportions of specific agreement and kappa's between the SRQ-20 and the EPDS within the Gambian cohort.

Table 2 shows the responses of the Gambian sample to the individual items on the SRQ-20. The somatic symptom items had a higher average score (*M* = 4.01, *SD* = 2.00) than psychological/cognitive/functional symptom items (*M* = 3.01, *SD* = 2.52). The somatic symptom items contributed more (63%) to the total SRQ-20 score than the psychological/cognitive/functional symptom items (37%).

For peer review only - http://bmjopen.bmj.com/site/about/guidelines.xhtml

Table 2. SRQ-20 Item, Item Category Frequency

SRQ20 Item		Yes *n* (%)
1	Do you often have headaches?	167 (76%)
2	Is your appetite poor?	121 (55%)
3	Do you sleep badly?	93 (42%)
4	Are you easily frightened?	82 (37%)
5	Do your hands shake?	24 (11%)
6	Do you feel nervous, tense or worried?	89 (40%)
7	Is your digestion poor?	78 (35%)
8	Do you have trouble thinking clearly?	58 (26%)
9	Do you feel unhappy?	94 (43%)
10	Do you cry more than usual?	20 (9%)
11	Do you find it difficult to enjoy your daily activities?	45 (20%)
12	Do you find it difficult to make decisions?	74 (33%)
13	Is your daily work suffering?	80 (36%)
14	Are you unable to play a useful part in life?	60 (27%)
15	Have you lost interest in things?	74 (33%)
16	Do you feel that you are a worthless person?	37 (17%)
17	Has the thought of ending your life been on your mind?	10 (5%)
18	Do you feel tired all the time?	116 (52%)
19	Do you have uncomfortable feelings in your stomach?	121 (55%)
20	Are you easily tired?	109 (49%)

Comparing EPDS scores in The Gambia and the UK

Figure 2 shows the distribution of total EPDS scores in the Gambian and UK samples. The UK average EPDS score ($M = 6.51$, $SD = 4.37$) was significantly higher than the Gambian average score ($M = 4.36$, $SD = 3.74$) ($p < 0.001$ 95%CIs [1.54, 2.84], $d = 0.53$). The UK scores were slightly positively skewed (skewness = 0.63), while the Gambian EPDS scores were highly positively skewed (skewness = 1.37), reflecting a higher frequency of lower scores in the Gambian sample.

Figure 2. EPDS distributions and cut-off score for UK and Gambia samples.

Cut-off Score Analysis

For peer review only

13% of UK participant's EPDS scores were ≥12 compared to 5% in the Gambian sample. With a cut-off of ≥ 10, 27% of UK versus 10% of Gambian participants were identified and when using the cut-off of ≥9, 34% UK versus 13% Gambian participants were identified (Figure 2). Thus, with each cut-off, two to three times more women scored above the selected cut-off score from the UK sample compared with the Gambian sample.

Item Analysis

Table 3 shows how the two samples scored on the individual items on the EPDS. In the Gambian sample the item scores were generally lower. The Gambian sample had a significantly higher average score (M = 0.41, SD = 0.61) compared to the UK sample (M = 0.25, SD = 0.52) only on the reverse coded item 2 (I have looked forward with enjoyment to things), (p < 0.004 95%CIs [0.26, 0.06], d = 0.28). For all other significant differences, the UK sample had a higher item average. The EPDS includes an anxiety subscale (EPDS-3) consisting of items 3, 4 and 5 [19]. This subscale average was also significantly higher for the UK sample (M = 3.49, SD = 2.03) compared with the Gambia sample (M = 1.7, SD = 1.67) (p < 0.004, 95%CIs [1.49, 2.09], d = 0.96). The anxiety subscale scores also contributed more (60%) to the total UK EPDS score, compared with the Gambia sample, where the subscale contributed to 39% of the total EPDS score. However, two of the most endorsed items in The Gambian sample were within the anxiety subscale (items 4 and 5).

For peer review only

Table 3. EPDS Item and Subscale Averages for the UK and Gambia Samples

		UK Sample	Gambia Sample
Item #		***M*(SD)**	***M*(SD)**
1	I have been able to laugh and see the funny side of things	0.19 (0.45)	0.28 (0.48)

For peer review only - http://bmjopen.bmj.com/site/about/guidelines.xhtml

2	I have looked forward with enjoyment to things*	0.25 (0.52)	0.41 (0.61)
3	I have blamed myself unnecessarily when things went wrong*	1.22 (0.78)	0.39 (0.65)
4	I have been anxious or worried for no good reason*	1.35 (0.87)	0.67 (0.80)
5	I have felt scared or panicky for no good reason*	0.92 (0.84)	0.64 (0.78)
6	Things have been getting on top of me*	0.98 (0.79)	0.64 (0.78)
7	I have been so unhappy that I have had difficulty sleeping	0.41 (0.69)	0.48 (0.79)
8	I have felt sad or miserable*	0.64 (0.67)	0.47 (0.66)
9	I have been so unhappy that I have been crying	0.47 (0.61)	0.33 (0.66)
10	The thought of harming myself had occurred to me	0.07 (0.31)	0.05 (0.26)
	**Total Score***	**6.51 (4.37)**	**4.36 (3.74)**

Note. Bonferroni adjusted alpha level of .004 was used per test (.05/13). **indicates a significant difference at $p < 0.004$*

For peer review only

Exploratory Factor Analysis

An exploratory factor analysis (EFA) was performed to investigate potential differences in the dimensionality of the EPDS between the two samples. Inspection of scree plots and the parallel analysis indicated the presence of 3 factors for both the UK and Gambia samples. However, the items that loaded onto these three factors were different (Table 4 and Figure 3). The UK Factor 1 was comprised of 6 items (questions 1, 2, 6, 7, 8, 9) that explained 33% of the variance, Factor 2 comprised 1 item (question 10) and explained 21% of the variance, and Factor 3 (questions 3, 4, 5 (the anxiety subscale)) consisted of 3 items and explained 17% of the variance. The total variance explained by all three factors was 72%. The Gambia Factor 1 was comprised of 5 items (questions 6, 7, 8, 9, 10) and explained 22% of the variance, Factor 2 was comprised of 3 items (questions 3, 4, 9) and explained 15% of the variance, and Factor 3 was comprised of two items (questions 1

For peer review only - http://bmjopen.bmj.com/site/about/guidelines.xhtml

and 2) and explained 9% of the variance. The total variance explained by all three factors was 47%.

Table 4. Factor Loadings for UK and Gambia Samples' EPDS Scores

**UK Sample**		**Factor 1**	**Factor 2**	**Factor 3**
Item				
1	I have been able to laugh and see the funny side of things	0.82		
2	I have looked forward with enjoyment to things	0.80		
3	I have blamed myself unnecessarily when things went wrong			0.69
4	I have been anxious or worried for no good reason			0.76
5	I have felt scared or panicky for no good reason			0.77
6	Things have been getting on top of me	0.54		
7	I have been so unhappy that I have had difficulty sleeping	0.75		
8	I have felt sad or miserable	0.80		
9	I have been so unhappy that I have been crying	0.71		
10	The thought of harming myself had occurred to me		1.41	
**Gambia Sample**		**Factor 1**	**Factor 2**	**Factor 3**
Item				
1	I have been able to laugh and see the funny side of things			0.41
2	I have looked forward with enjoyment to things			0.79
3	I have blamed myself unnecessarily when things went wrong		0.99	
4	I have been anxious or worried for no good reason		0.41	
5	I have felt scared or panicky for no good reason			
6	Things have been getting on top of me	0.62		
7	I have been so unhappy that I have had difficulty sleeping	0.69		
8	I have felt sad or miserable	0.52		
9	I have been so unhappy that I have been crying	0.53	0.41	

For peer review only

For peer review only - http://bmjopen.bmj.com/site/about/guidelines.xhtml

10	The thought of harming myself had occurred to me	0.85

Note: Factor loadings < .4 are suppressed as suggested by [28].

Figure 3. UK and Gambia samples' EPDS factor structure. The third factor of the UK sample's EFA includes the same three items found to make up the EPDS-3 anxiety subscale.

DISCUSSION

When comparing scores on the EPDS between the Gambian and UK samples, the UK participants had significantly higher EPDS scores; 13% of UK participant's scores were ≥12 compared with 5% of the Gambian sample. This could, of course, be taken at face value to indicate higher levels of CMS symptoms in the UK population. However, the comparison of EPDS and SRQ-20 scores reveals an alternative view. While only 5% of Gambian participants met threshold for high levels of symptoms with EPDS, that number rose to 42% when the SRQ-20 was used. Thus, the EPDS seems to significantly underestimates the incidence of CMD symptoms in this Gambian sample. This may be due to the fact that the EPDS does not capture the somatic way in which CMD symptoms are experienced.

The question of why somatic symptoms, rather than those of depressive mood, were more frequently endorsed by our Gambian sample, may be in part because of differences of language. Different ways of describing the world can lead speakers of different languages to have different ways of thinking about the world [29]. Differences in CMD symptom presentation when measured using translated self-report measures may not reflect Gambian indigenous understandings of symptom causation. These indigenous understandings may contrast with biomedical perspectives [30]. For example, a systematic review found that local language versions of the EPDS in LMICs

For peer review only

For peer review only - http://bmjopen.bmj.com/site/about/guidelines.xhtml

had lower precision for identifying true cases of perinatal CMDs compared to the original English version [31]. Additionally, Molenaar et al. [32] found that in a perinatal population in rural Ethiopia, most women recognised the existence of perinatal mental distress states, but did not call such distress a discrete illness, such as depression. Instead, these mental distress states were generally seen as non-pathological reactions to difficult circumstances.

Some researchers have raised the concern that measuring somatic CMD symptoms in populations with high levels of physical disease might mistakenly attribute these symptoms to a depressive syndrome[12]. In a perinatal population this may present an even more difficult problem as somatic symptoms related to pregnancy are expected to be experienced and change throughout this time. However, an important study by Stewart et al. [9], found that the inclusion of somatic items did not affect the test performance of the SRQ-20 in their Malawi postnatal population as validated by a semi-structured diagnostic interview for depressive disorder.

Previous research has found that somatization of mental health disorders in general, and perinatal disorders in particular, is common in African populations [5,10,33]. This supports the idea that in many non-western cultures the symptoms of dysphoric disorders are mostly somatic and are different from the Western systems [34]. This current study is in agreement with previous research that used the SRQ-20 in other perinatal African populations and found the somatic items from the SRQ-20 were, indeed, the most frequently endorsed [9,10].

Additionally, the EPDS showed a different factor structure for the two samples. While the factor structure identified from the UK sample’s scores was similar to that identified within other studies in the UK and other HICs, with the clear identification of the anxiety subscale [35], the structure identified from the Gambian sample’s scores was different (three-factors) and accounted for less of the variability in the total scores. In the UK sample Factor 1 included many of the items

For peer review only

For peer review only - http://bmjopen.bmj.com/site/about/guidelines.xhtml

related to sadness or crying. These items were some of the least endorsed by the Gambian participants. Item 2 (I have looked forward with enjoyment to things) was the only item where the average score of the Gambian sample was significantly higher than the average score of the UK sample. It may be that the idea of anticipating or expecting enjoyment in your future might go against ideas of humility and patience, and faith in God (any plans for the future are normally concluded with 'inshallah', God willing). The results of the exploratory factor analysis from the Gambian sample display a different factor structure to previous work in HICs [35], and also to other countries in Africa (e.g., South Africa where all items loaded onto one factor [36]). Taken together, as has been found in previous studies [3,37,38], our results further emphasise how methods and understanding around measuring perinatal mental health symptoms developed in Western countries need to employed with care when working across different cultures.

For peer review only

Limitations

How the EPDS was used was different in the two settings. The EPDS was self-completed in written form in the UK versus delivered and responded to orally in The Gambia due to the low literacy rate in the population. Other factors include differences in the recruitment process, purpose and setting (hospital versus community), and in demographic factors such as age and parity. Therefore, these other factors might explain, in part, some of the differences reported on the EPDS between participants in The Gambia versus the UK. However, it seems unlikely that these factors alone could explain the major differences in response.

The SRQ-20's binomial response format, compared to the EPDS Likert-scale response format, may have influenced the differences observed in the performance of these two tools. The binomial response format is more easily administered and understood by respondents with low

For peer review only - http://bmjopen.bmj.com/site/about/guidelines.xhtml

literacy [38]. Responses like ‘hardly ever’ and ‘sometimes’ might have been difficult to distinguish. A study comparing these two scales in a Ghanaian pregnant population found that it was important to alter the format of the EPDS in Ghana, for it to be understandable to women [39]. Additionally, while careful consideration and translation of the SRQ-20 and EPDS helped to ensure semantic validity of the scales for use in The Gambia, the scales have not been validated against a clinical gold-standard (clinical interview) to screen for possible CMD, highlighting a potential limitation of this work.

CONCLUSION

In conclusion, these findings support studies conducted in other parts of Africa [9,10,40], adding to the literature by exploring differences within a new cultural context. It will be of interest in the future to explore further the cultural reasons for such discrepancies. This study helps to further underline the importance of investigating differences in scale performance within and across cultures to ensure that the measurement scale chosen is sensitive to cultural differences in the understanding and presentation of perinatal common mental disorders.

Funding

This study was funded by the MRC-AHRC Global Public Health: Partnership Awards scheme (MR/R024618/1) awarded to Professor Lauren Stewart. This writing of this manuscript was supported by a South East Network for Social Sciences/The Economic and Social Research Council funded postdoctoral fellowship awarded to Dr Katie Rose Sanfilippo (Grant Reference Number: ES/V010158/1).

For peer review only

For peer review only - http://bmjopen.bmj.com/site/about/guidelines.xhtml

Competing interests

The authors declare no competing interests

Author Contributions

The manuscript was prepared by KRMS and all authors approved the final draft. KRMS and BM helped with data management and data collection materials. BD provided local knowledge, expertise in health provision and healthcare in The Gambia, and organisation in the field liaising with antenatal clinics. HC provided management of the funds and transportation in The Gambia. HBH and MG completed all data collection and input and provided knowledge of mental health and reproductive health in the Gambia. KRMS completed data cleaning and analysis. VC provided methodological and statistical expertise. VG and PR provided expertise in perinatal mental health. IC provided expertise on culture and language and BM, HC and BD provided expertise on Gambian culture and language. RAC was responsible for all data collection and management for the UK sample. All authors, except V, RAC and IC, spent time in The Gambia supervising the project on the ground. LS is the grant holder and principal investigator and oversaw all aspects of the study.

Data sharing statement

De-identified participant data that correspond to the results reported in this article are available upon reasonable request from the corresponding author (katie-rose.sanfilippo@city.ac.uk).

Ethical approval and consent to participate

For peer review only

For peer review only - http://bmjopen.bmj.com/site/about/guidelines.xhtml

All procedures performed in studies involving human participants were in accordance with the ethical standards of the institutional and/or national research committee and with the 1964 Helsinki Declaration and its later amendments or comparable ethical standards. All procedures involving human participants were approved by the Goldsmiths University Ethics Committee (1392), The Gambia Government/MRC Gambia joint Ethics Committee (R018 015V2) and the Australian National University Ethics Committee (2018/235). Members of the research team carried out the consenting and conduct of this study orally. It was emphasised that any participant was able to withdraw from the study at any point without any consequences.

For peer review only

Acknowledgements

We would like to thank the women and clinicians in the antenatal clinics in the Gambia and at Queen Charlotte's and Chelsea Hospital in the UK. Thank you also to Pa Bakary Sonko and Charlotte Hanlon for their advice and support, Giles Partington for help with analysis advice and Jane Offerman for helping with administration.

REFERENCES

1 Glover V, O'Donnell KJ, O'Connor TG, *et al.* Prenatal maternal stress, fetal programming, and mechanisms underlying later psychopathology - A global perspective. *Development and Psychopathology* 2018;**30**:843–54. doi:10.1017/S095457941800038X

2 Fisher J, de Mello MC, Patel V, *et al.* Prevalence and determinants of common perinatal mental disorders in women in low- and lower-middle-income countries: A systematic review. *Bull World Health Organ* 2012;**90**:139–49. doi:10.2471/BLT.11.091850

3 Kirmayer LJ. Cultural Variations in the Clinical Presentation of Depression and Anxiety: Implications for Diagnosis and Treatment. *J Clin Psychiatry* 2001;**62**:22–8.

4 Oates MR, Cox JL, Neema S, *et al.* Postnatal depression across countries and cultures: A qualitative study. *British Journal of Psychiatry* 2004;**184**:s10–6.

5 Senturk V, Hanlon C, Medhin G, *et al.* Impact of perinatal somatic and common mental disorder symptoms on functioning in Ethiopian women: The P-MaMiE population-

For peer review only - http://bmjopen.bmj.com/site/about/guidelines.xhtml

based cohort study. *Journal of Affective Disorders* 2012;**136**:340–9. doi:10.1016/j.jad.2011.11.028

6 Cox JL, Holden J, Henshaw C. *Perinatal mental health: The Edinburgh Postnatal Depression Scale (EPDS) manual*. 2nd ed. London, UK: : The Royal College of Psyciatrists Publications 2014.

7 Ali GC, Ryan G, De Silva MJ. Validated screening tools for common mental disorders in low and middle income countries: A systematic review. *PLoS ONE* 2016;**11**:e0156939. doi:10.1371/journal.pone.0156939

8 Beusenberg M, Orley J. A user's guide to the Self Reporting Questionnaire (SRQ). 1994.

9 Stewart RC, Kauye F, Umar E, *et al.* Validation of a Chichewa version of the Self-Reporting Questionnaire (SRQ) as a brief screening measure for maternal depressive disorder in Malawi, Africa. *Journal of Affective Disorders* 2009;**112**:126–34. doi:10.1016/j.jad.2008.04.001

10 Hanlon C, Medhin G, Alem A, *et al.* Detecting perinatal common mental disorders in Ethiopia: Validation of the self-reporting questionnaire and Edinburgh Postnatal Depression Scale. *Journal of Affective Disorders* 2008;**108**:251–62. doi:10.1016/j.jad.2007.10.023

11 Okulate GT, Olayinka MO, Jones OBE. Somatic symptoms in depression: Evaluation of their diagnostic weight in an African setting. *British Journal of Psychiatry* 2004;**184**:422–7. doi:10.1192/bjp.184.5.422

12 Cox JL, Holden JM, Sagovsky R. Detection of postnatal depression: Development of the 10-item Edinburgh Postnatal Depression Scale. *The British Journal of Psychiatry* 1987;**150**:782–6. doi:10.1192/bjp.150.6.782

13 Kozinszky Z, Dudas RB. Validation studies of the Edinburgh Postnatal Depression Scale for the antenatal period. *Journal of Affective Disorders* 2015;**176**:95–105. doi:10.1016/j.jad.2015.01.044

14 Tsai AC, Scott JA, Hung KJ, *et al.* Reliability and validity of instruments for assessing perinatal depression in African settings: Systematic review and meta-analysis. *PLoS ONE* 2013;**8**:e82521. doi:10.1371/journal.pone.0082521

15 Coleman R, Morison L, Paine K, *et al.* Women's reproductive health and depression: A community survey in The Gambia, West Africa. *Social Psychiatry and Psychiatric Epidemiology* 2006;**41**:720–7. doi:10.1007/s00127-006-0085-8

16 Nabwera HM, Moore SE, Mwangome MK, *et al.* The influence of maternal psychosocial circumstances and physical environment on the risk of severe wasting in rural Gambian infants: A mixed methods approach. *BMC Public Health* 2018;**18**:109. doi:10.1186/s12889-017-4984-2

17 Sanfilippo KRM, McConnell B, Cornelius V, *et al.* Community psychosocial music intervention (CHIME) to reduce antenatal common mental disorder symptoms in The Gambia: a feasibility trial. *BMJ Open* 2020;**10**:e040287. doi:10.1136/bmjopen-2020-040287

18 Bartram C. *Early infant social behaviour and maternal perinatal depression in Kiang West , The Gambia*. 2018.

19 Mitchell AJ. The 3 item anxiety subscale of the Edinburgh Postpartum Depression Scale may detect postnatal depression as well as the 10 item full scale. *Evidence-Based Mental Health* 2009;**12**:44. doi:10.1136/ebmh.12.2.44

For peer review only - http://bmjopen.bmj.com/site/about/guidelines.xhtml

20 Sawyer A, Ayers S, Smith H, *et al.* Women's experiences of pregnancy, childbirth, and the postnatal period in the Gambia: A qualitative study. *British Journal of Health Psychology* 2011;**16**:528–41. doi:10.1348/135910710X528710

21 World Health Organization. Process of translation and adaptation of instruments. 2014.

22 The Gambia Bureau of Statistics (GBOS) and ICF International. The Gambia Demographic and Health Survey 2013. Banjul, The Gambia and Rockville, Maryland, USA: 2014.

23 Sanfilippo KRM, McConnell B, Cornelius V, *et al.* A study protocol for testing the feasibility of a randomised stepped wedge cluster design to investigate a Community Health Intervention through Musical Engagement (CHIME) for perinatal mental health in The Gambia. *Pilot and Feasibility Studies* 2019;**5**:124. doi:10.1186/s40814-019-0515-5

24 R Core Team. R: A language and environment for statistical computing. 2013.

25 Martin Bland J, Altman DG. Statistical methods for assessing the agreement between two methods of clinical measurement. *The Lancet* 1986;**327**:307–10. doi:10.1016/S0140-6736(86)90837-8

26 Cicchetti D V., Feinstein AR. High agreement but low kappa: II. Resolving the paradoxes. *Journal of Clinical Epidemiology* 1990;**43**:551–8. doi:10.1016/0895-4356(90)90159-M

27 Baglin J. Improving your exploratory factor analysis for ordinal data: A demonstration using FACTOR. *Practical Assessment, Research & Evaluation* 2014;**19**.

28 Field A. *Discovering statistics using SPSS*. 3rd ed. SAGE Publications Ltd 2009.

29 Boroditsky L. Linguistic Relativity. In: Nadel L, ed. *Encyclopedia of Cognitive Science*. Chichester: : John Wiley & Sons, Ltd 2006. doi:10.1002/0470018860.s00567

30 McConnell BB. *Music, health, and power: Singing the unsayable in The Gambia*. New York, NY: : Routledge 2020. doi:10.4324/9780367312732

31 Shrestha SD, Pradhan R, Tran TD, *et al.* Reliability and validity of the Edinburgh Postnatal Depression Scale (EPDS) for detecting perinatal common mental disorders (PCMDs) among women in low-and lower-middle-income countries: A systematic review. *BMC Pregnancy and Childbirth* 2016;**16**:1–19. doi:10.1186/S12884-016-0859-2/FIGURES/3

32 Molenaar J, Hanlon C, Alem A, *et al.* Perinatal mental distress in a rural Ethiopian community: A critical examination of psychiatric labels. *BMC Psychiatry* 2020;**20**. doi:10.1186/s12888-020-02646-5

33 Patel V. *Culture and common mental disorders in sub-Saharan Africa*. East Sussex, UK: : Psychology Press 1998. doi:10.4324/9781315826455

34 Halbreich U, Karkun S. Cross-cultural and social diversity of prevalence of postpartum depression and depressive symptoms. *Journal of Affective Disorders* 2006;**91**:97–111. doi:10.1016/j.jad.2005.12.051

35 Matthey S, Fisher J, Rowe H. Using the Edinburgh postnatal depression scale to screen for anxiety disorders: Conceptual and methodological considerations. *Journal of Affective Disorders* 2013;**146**:224–30. doi:10.1016/j.jad.2012.09.009

36 De Bruin GP, Swartz L, Tomlinson M, *et al.* The factor structure of the Edinburgh Postnatal Depression scale in a South African peri-urban settlement. *South African Journal of Psychology* 2004;**34**:113–21. doi:10.1177/008124630403400107

For peer review only - http://bmjopen.bmj.com/site/about/guidelines.xhtml

37 Kortmann F. Problems in communication in transcultural psychiatry: The self reporting questionnaire in Ethiopia. *Acta Psychiatrica Scandinavica* 1987;**75**:563–70. doi:10.1111/j.1600-0447.1987.tb02836.x

38 Sweetland AC, Belkin GS, Verdeli H. Measuring depression and anxiety in sub-Saharan Africa. *Depression and Anxiety* 2014;**31**:223–32. doi:10.1002/da.22142

39 Weobong B, Akpalu B, Doku V, *et al.* The comparative validity of screening scales for postnatal common mental disorder in Kintampo, Ghana. *Journal of Affective Disorders* 2009;**113**:109–17. doi:10.1016/j.jad.2008.05.009

40 Chorwe-Sungania G, Chipps J. Validity and utility of instruments for screening of depression in women attending antenatal clinics in Blantyre district in Malawi. *South African Family Practice* 2018;**60**:55. doi:10.4102/safp.v60i4.4888

For peer review only

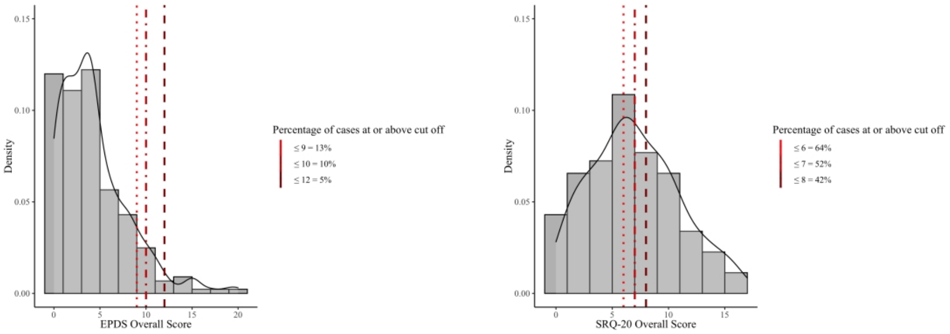

Histogram of different cut-off scores for EPDS and SRQ-20.

1587x529mm (72 x 72 DPI)

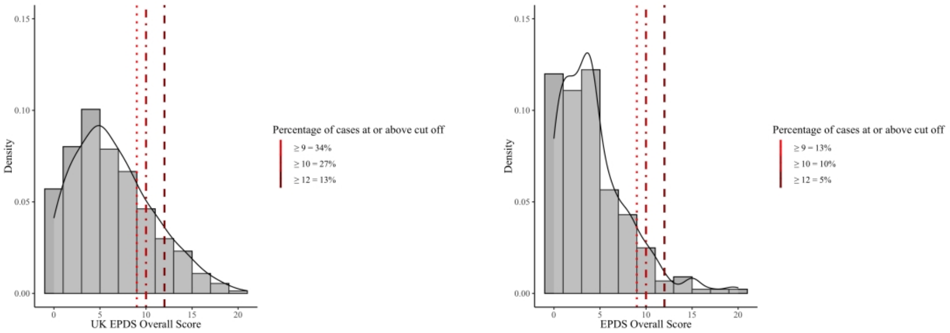

EPDS distributions and cut-off score for UK and Gambia samples.

1587x529mm (72 x 72 DPI)

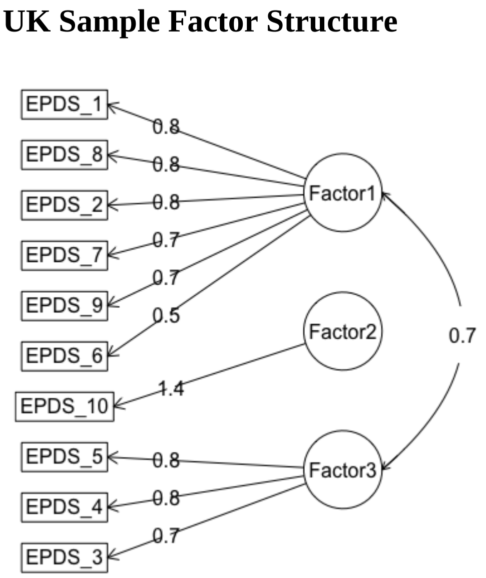

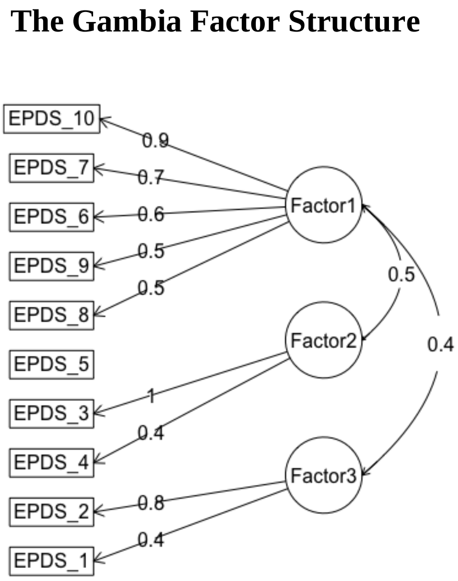

For peer review only

Supplementary Material 1

Table sup 1 Antenatal Clinic Names, Type, Area and Language

**Antenatal Clinic**	**Clinic Type**	**Area Type**	**Language**
Banjuliding Health Centre	Minor Health Clinic	Urban	Mandinka
Essau Distract Hospital	District hospital	Urban	Wolof
Fajikunda Health Centre	Major Health Clinic	Urban	Wolof
Gunjur Health Centre	Minor Health Clinic	Rural	Mandinka
Kafuta Health Centre	Minor Health Clinic	Rural	Mandinka
Kuntair Health Centre	Minor Health Clinic	Rural	Wolof
Pirang Health Centre	Minor Health Clinic	Rural	Mandinka
Serekunda Health Centre	Major Health Clinic	Urban	Wolof
Sinchu Baliya Health Post	Community Clinic/Health Post	Urban	Wolof
Sukuta Health Centre	Minor Health Clinic	Urban	Mandinka

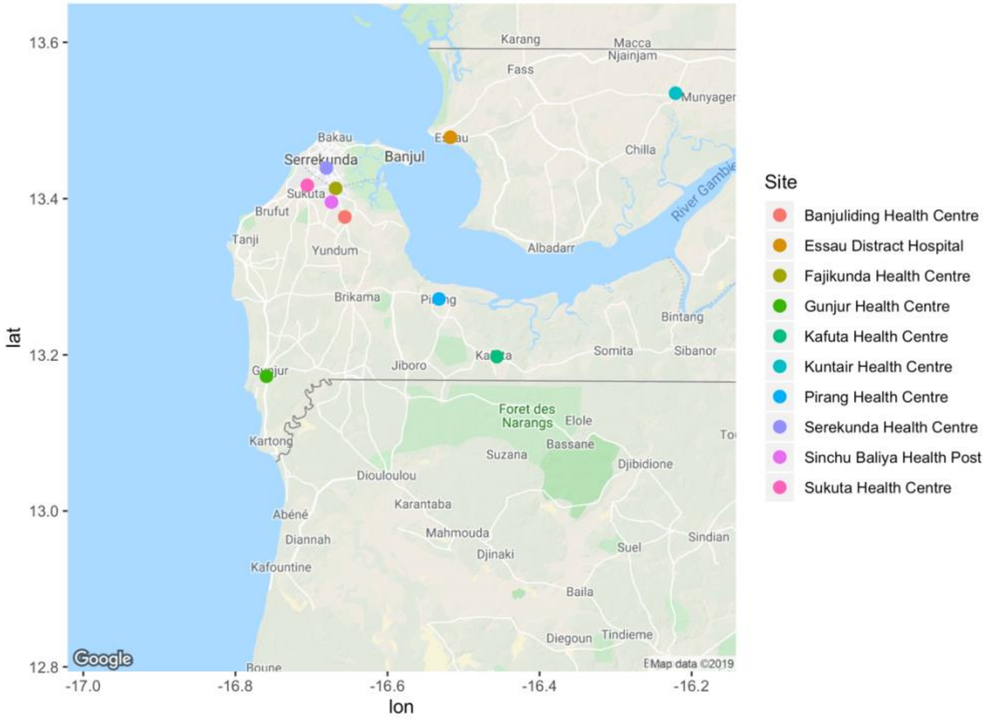

Fig sup 1. A map of the participating antenatal clinics described in Table sup 1.

Supplementary Materials 3

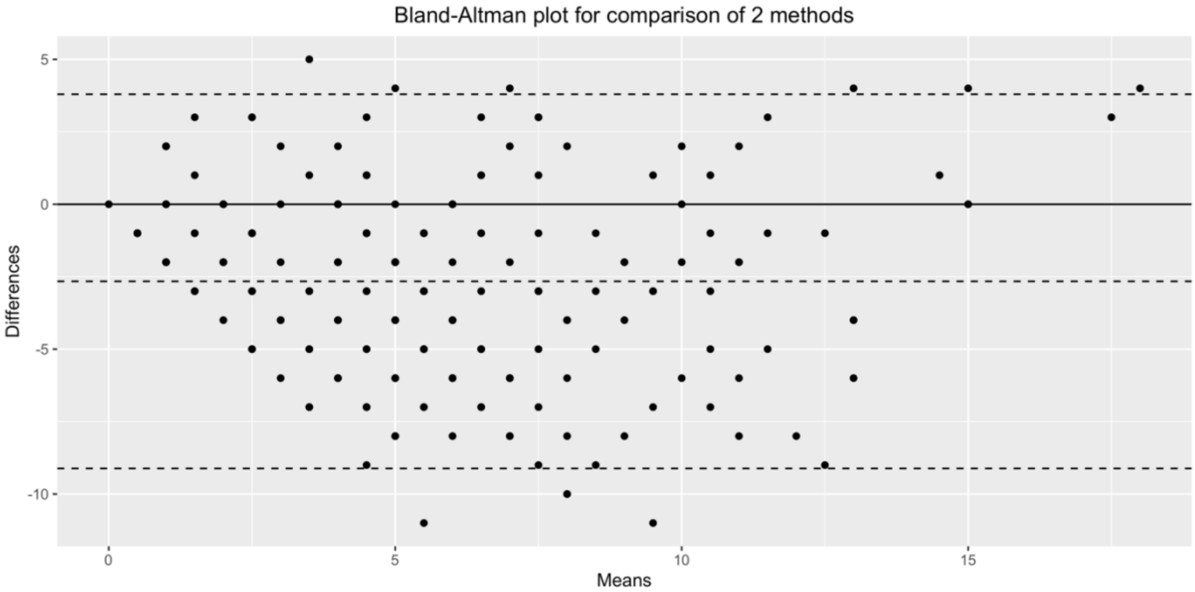

Fig Sup.3. Bland-Altman Plot comparing EPDS and SRQ-20 scores within the Gambian pregnant sample.

Supplementary Materials 4

Table Sup.4.

Proportions of Specific Agreement and Kappa's

Cut-offs compared	Positive Agreement *n* (%)	Negative Agreement *n* (%)	Overall Agreement *n* (%)	Dis-agreement *n* (%)	Cohen's Kappa
EPDS ≥ 12 & SRQ20 ≥ 8	10 (19%)	128 (76%)	138 (60%)	83 (36%)	0.12
EPDS ≥ 12 & SRQ20 ≥ 7	10 (16%)	107 (67%)	117 (51%)	104 (45%)	0.09
EPDS ≥ 12 & SRQ20 ≥ 6	10 (13%)	80 (55%)	90 (39%)	131 (57%)	0.05
EPDS ≥ 10 & SRQ20 ≥ 8	21 (37%)	128 (78%)	149 (65%)	72 (31%)	0.25
EPDS ≥ 10 & SRQ20 ≥ 7	21 (31%)	107 (70%)	128 (56%)	93 (41%)	0.18
EPDS ≥ 10 & SRQ20 ≥ 6	21 (26%)	80 (57%)	101 (44%)	120 (52%)	0.11
EPDS ≥ 9 & SRQ20 ≥ 8	24 (40%)	124 (77%)	148 (65%)	73 (32%)	0.25
EPDS ≥ 9 & SRQ20 ≥ 7	25 (35%)	104 (69%)	129 (56%)	92 (40%)	0.19
EPDS ≥ 9 & SRQ20 ≥ 6	27 (32%)	79 (58%)	106 (46%)	115 (50%)	0.14
**Average Agreement**	**28%**	**67%**	**54%**	**43%**	

Note. Percent positive agreement = 2(number both ≥ cut off)/2(number both ≥ cut off) + total number of disagreement; Percent negative agreement = 2(number both ≤ cut off)/2(number both ≤ cut off) + total number of disagreement (Cicchetti & Feinstein, 1990)

For peer review only

BMJ Open

Expression of Antenatal Symptoms of Common Mental Disorders in The Gambia and the UK: A cross-sectional correlation comparison study

Journal:	*BMJ Open*
Manuscript ID	bmjopen-2022-066807.R1
Article Type:	Original research
Date Submitted by the Author:	03-May-2023

Complete List of Authors:	Sanfilippo, Katie Rose; City University of London, Centre for Healthcare Innovation Research<br>Glover, Vivette; Imperial College London<br>Cornelius, Victoria; Imperial College London, Health and Social Care Research<br>Castro , Rita ; Universitat Zurich, Psychologisches Institut<br>McConnell, Bonnie ; Australian National University,<br>Darboe, Buba; Ministry of Health and Social Welfare The Gambia<br>Huma, Hajara; Ministry of Health and Social Welfare The Gambia; National Centre for Arts and Culture The Gambia<br>Ceesay, Hassoum ; National Centre for Arts and Culture<br>Ramchandani, Paul; University of Cambridge, Education<br>Cross, Ian; Cambridge University<br>Stewart, Lauren ; Goldsmiths University of London

**Primary Subject Heading**:	Mental health
Secondary Subject Heading:	Global health, Obstetrics and gynaecology
Keywords:	MENTAL HEALTH, Depression & mood disorders < PSYCHIATRY, Anxiety disorders < PSYCHIATRY

SCHOLARONE™
Manuscripts

I, the Submitting Author has the right to grant and does grant on behalf of all authors of the Work (as defined in the below author licence), an exclusive licence and/or a non-exclusive licence for contributions from authors who are: i) UK Crown employees; ii) where BMJ has agreed a CC-BY licence shall apply, and/or iii) in accordance with the terms applicable for US Federal Government officers or employees acting as part of their official duties; on a worldwide, perpetual, irrevocable, royalty-free basis to BMJ Publishing Group Ltd ("BMJ") its licensees and where the relevant Journal is co-owned by BMJ to the co-owners of the Journal, to publish the Work in this journal and any other BMJ products and to exploit all rights, as set out in our licence.

The Submitting Author accepts and understands that any supply made under these terms is made by BMJ to the Submitting Author unless you are acting as an employee on behalf of your employer or a postgraduate student of an affiliated institution which is paying any applicable article publishing charge ("APC") for Open Access articles. Where the Submitting Author wishes to make the Work available on an Open Access basis (and intends to pay the relevant APC), the terms of reuse of such Open Access shall be governed by a Creative Commons licence – details of these licences and which Creative Commons licence will apply to this Work are set out in our licence referred to above.

Other than as permitted in any relevant BMJ Author's Self Archiving Policies, I confirm this Work has not been accepted for publication elsewhere, is not being considered for publication elsewhere and does not duplicate material already published. I confirm all authors consent to publication of this Work and authorise the granting of this licence.

For peer review only

Expression of Antenatal Symptoms of Common Mental Disorders in The Gambia and the UK: A cross-sectional correlation comparison study

Katie Rose M. Sanfilippo[1], Vivette Glover[2], Victoria Cornelius[3], Rita Amiel Castro[4], Bonnie McConnell[5,] Buba Darboe[6], Hajara B. Huma[6,7], Hassoum Ceesay[7], Paul Ramchandani[8] , Ian Cross[9], Lauren Stewart[10]

[1]*Centre for Healthcare Innovation Research, City, University of London, UK*

[2]*Institute of Reproductive and Developmental Biology, Imperial College London, UK*

[3]*Imperial Clinical Trails Unit, School of Public Health, Imperial College London, UK*

[4] *Department of Clinical Psychology and Psychotherapy, Institute of Psychology, University of Zurich, Switzerland*

[5]*School of Music, Australian National University, Canberra, Australia*

[6]*The Ministry of Health and Social Welfare, The Gambia*

[7]*The National Centre for Arts and Culture, The Gambia*

[8]*Faculty of Education, University of Cambridge, UK*

[9]*Faculty of Music, University of Cambridge, UK*

[10]*Psychology Department, Goldsmiths, University of London, UK*

Correspondence concerning this paper should be addressed to Katie Rose M. Sanfilippo City, University of London at katie-rose.sanfilippo@city.ac.uk

Word Count: 4,174

For peer review only

For peer review only - http://bmjopen.bmj.com/site/about/guidelines.xhtml

ABSTRACT

Objectives

It is important to be able to detect symptoms of common mental disorders (CMDs) in pregnant women. However, the expression of these disorders can differ across cultures and depend on the specific scale used. This study aimed to (a) compare Gambian pregnant women's responses to the Edinburgh Postnatal Depression Scale (EPDS) and Self-reporting Questionnaire (SRQ-20) and (b) compare responses to the EPDS in pregnant women in The Gambia and UK.

Design

This cross-sectional correlation comparison study investigates Gambian EPDS and SRQ-20 scores through correlation between the two scales, score distributions, the proportion of women with high levels of symptoms, and descriptive item analysis. Comparisons between the UK and Gambian EPDS scores were made by investigating score distributions, the proportion of women with high levels of symptoms, and descriptive item analysis.

Setting

This study took place in The Gambia, West Africa and London, UK.

Participants

221 pregnant women from The Gambia completed both the SRQ-20 and the EPDS. 368 pregnant women from the UK completed the EPDS.

Results

Gambian participants' EPDS and SRQ-20 scores were significantly moderately correlated (r_s = 0.6, $p < 0.00$), had different distributions, 54% overall agreement, and different proportions of women identified as having high levels of symptoms (SRQ-20 = 42% versus EPDS = 5% using highest cut-off score). UK participants had higher EPDS scores (*M* = 6.5, 95%CI[6.1, 6.9]) than

For peer review only - http://bmjopen.bmj.com/site/about/guidelines.xhtml

the Gambian participants (*M* = 4.4, 95% CI[3.9, 4.9]) ($p < 0.001$, 95%CIs [-3.0, -1.0], *Cliff's delta* = - 0.3).

Conclusions

The differences in scores from Gambian pregnant women to the EPDS and SRQ-20 and the different EPDS responses between pregnant women in the UK and The Gambia further emphasise how methods and understanding around measuring perinatal mental health symptoms developed in Western countries need to be applied with care in other cultures.

Keywords:

Perinatal; Common Mental Disorders; EPDS; SRQ-20; The Gambia; United Kingdom

ARTICLE SUMMARY

Strengths and limitations of this study

- Findings support studies conducted in other parts of Africa, adding to the literature by exploring differences within a new cultural context: The Gambia.
- This study compares two different scales (EPDS and SRQ-20) within the same cultural context in addition to comparing one scale (EPDS) across cultures.
- This study includes a relatively large sample size across The Gambia collected across 10 antenatal clinics across the West Coast and North Bank regions.
- EPDS and SRQ-20 have not been validated in The Gambia against a clinical gold-standard (clinical interview) to screen for possible CMD.

BACKGROUND

For peer review only - http://bmjopen.bmj.com/site/about/guidelines.xhtml

For peer review only

It is important to be able to recognise and treat symptoms of common mental disorders (CMDs), including depression and anxiety, during pregnancy. These symptoms affect not only the mother but can also have long-term adverse effects on her child [1]. Perinatal mental health problems are a particular challenge in low- and middle-income countries (LMICs) where they can be at least twice as frequent as in higher-income countries and are often associated with stigma [2]. Thus, it is necessary to be able to identify these disorders both for intervention and research purposes. Short, simple self-rating questionnaires are often the easiest way to do this. However, women in different cultures and settings can have a different experience and presentation of CMD symptoms which may lead to under recognition or misidentification of psychological distress [3], impacting where women might seek support and what treatment options they are offered, if any. For instance, Oates et al [4], in a qualitative cross-cultural study of postnatal depression, found that while all cultures described a condition of 'morbid unhappiness' after childbirth, not everyone saw it as an illness remediable by health interventions. Likewise, the presentation of symptoms is not uniform across different populations. Previous work in different African populations have suggested that CMD symptoms are experienced in a more somatic way[5].

The EPDS, a commonly used tool to measure CMD symptoms, deliberately minimizes items of a somatic nature since it has been argued that physical changes in pregnancy might confound interpretation of CMD symptoms [6]. Even so, it has been recommended specifically for perinatal depression detection in LMICs [7]. However, Cox, Holden and Henshaw [6] note that the use of the EPDS in certain cultures may be inappropriate. Given that some populations experience CMD symptoms in a more somatic way, the use of a scale which precludes their detection is likely to underestimate the incidence of CMD symptoms in such populations. An alternative to the EPDS is the Self Reporting Questionnaire (SRQ-20)[8], developed by the World

For peer review only - http://bmjopen.bmj.com/site/about/guidelines.xhtml

Health Organisation for the detection of symptoms of common mental disorders in LMICs. It is suggested to be useful for measuring perinatal CMDs in Africa due to its inclusion of somatic symptom items [9,10].

While the somatization of CMDs has been documented in a number of African populations [5,10,11], the existing bias for research studies to concentrate on western populations, as well as the diversity of the African continent, motivates exploring this question in different populations and with different methodological approaches. In the current study we compare how pregnant women of Gambian nationality (living in the Gambia) scored on two common measures of CMD symptoms, the EPDS and the SRQ-20. We also compare CMD symptoms, as measured by the EPDS, of these Gambian women against those of UK nationals (living in London, UK). Therefore, the objectives of this study were to explore differences between two scales within the same cultural context (The Gambia) and one scale across two different contexts (The UK and The Gambia).

METHODS

This study is a cross-sectional correlation comparison study where we use a comparative and descriptive analysis approach. This is not a validation study but rather exploratory and descriptive in nature to compare how two different scales used to measure perinatal CMD symptoms perform within The Gambia and, also, how the same scale (EPDS) performs across two different countries. For this paper the STROBE guidelines were followed, and the checklist can be found as Supplementary Material 1.

Participants and Setting

For peer review only - http://bmjopen.bmj.com/site/about/guidelines.xhtml

A convenience sample of 221 Gambian pregnant women between 10-30 weeks gestation (145 = Mandinka speaking and 76 = Wolof speaking) were assessed from May – August 2018. This was a sufficient sample size as it was determined that a sample size of 194 was needed to detect a correlation coefficient of 0.2 and a sample size of 124 was needed for a cross-sectional study design to detect a prevalence of 26% (the estimate of prevalence of antenatal depression for women in LMICs[12]) when using a two-sided correlation test, 5% significance level test (α=0.05) with 80% power (β=0.2) [13,14].

The Gambia is a country on the West African coast and is made up of eight different ethnic groups all with their own languages and culture. Islam is the predominant religion in The Gambia and is a powerful factor in shaping life, beliefs, and expectations. Participants were recruited from 10 different antenatal clinics across the western part of The Gambia (for more information see Supplementary Material 2). Any woman who attended the clinic, was above 18 years old and spoke either Mandinka or Wolof was approached. Women were excluded if they had previous or current psychosis or a history of a late term miscarriage.

For the second part of the study, existing data from a convenience sample of 368 pregnant women living in the UK from Queen Charlotte's and Chelsea Hospital in London was used. This data was part of a previous longitudinal study where women were recruited from April 2013 to April 2014. Exclusion criteria was therefore different to that of the Gambian cohort and excluded participants who did not speak and/or write English, did not have a device with internet access, had a multiple pregnancy, in vitro fertilization, severe medical problems, pregnancy medical problems (including abnormal foetus), or severe psychiatric problems (e.g. psychosis, suicidality or drug addiction).

For peer review only - http://bmjopen.bmj.com/site/about/guidelines.xhtml

Measurement scales

The EPDS [15] is a ten-item scale that was designed to measure postnatal depression and anxiety symptoms that has subsequently been validated for antenatal use [16]. It has been used with perinatal populations across Africa [17] and in perinatal populations in The Gambia [18–21], though a validated version in Mandinka or Wolof could not be obtained. Participants answer on a four-point Likert-scale (from 0 – 3) how often they have experienced a specific symptom within the last week. The three anxiety items (items 3, 4 and 5) have been shown to form a valid anxiety subscale (EPDS-3 [22]). A higher total score (out of a total of 30) corresponds to a higher level of symptoms.

The SRQ-20 [8] is a 20-item scale that measures symptoms of CMDs. It was developed to be used in primary care settings in LMICs and has also been used in perinatal populations across Africa [9,10,23] and The Gambia [21]though, in The Gambia, it has not been validated against a gold-standard clinical interview. The SRQ-20 includes items measuring common somatic symptoms associated with anxiety and depression (headaches, low appetite, poor digestion, and sleep problems) (items 1–3, 5, 7, 18–20) as well as other psychological and physical/somatic symptoms (feeling frightened, unhappy, worthless, and low-energy) (items 4, 6, 8, 9–17) [8,10]. To each item, participants answer either yes = 1 or no = 0. The scores range from 0 – 20 with a higher score indicating higher levels of CMD symptoms.

Both scales were translated into Mandinka and Wolof using methods based on recommendations from the WHO [24], Hanlon et al., [25], and Cox, Holden and Henshaw [6]. First, the scales were translated into Mandinka and Wolof by bilingual experts. The terms used and concepts around mental distress were informed by a large qualitative study held prior to this work. An expert panel, consisting of experts in Gambian culture and language and an expert in

For peer review only - http://bmjopen.bmj.com/site/about/guidelines.xhtml

mental health from the Ministry of Health and Social Welfare, held a discussion and then refined the translation. Both were then back-translated into English and reviewed again by the expert panel that developed the final version.

Procedure

Local midwives in The Gambia identified possible participants. If participants met the inclusion criteria, they were given information about the study by the two research assistants (RAs). Both RAs were trained psychiatric nurses and one was also a midwife. Verbal informed consent was obtained, witnessed, and recorded via RA signature or participant thumbprint. Due to low levels of literacy in The Gambia[26], participants' EPDS and SRQ-20 responses were collected orally by the RAs in alternating order per participant. There were no order effects found when using an independent samples t-test ($p > 0.05$, CIs [4.37, 4.30]). The RAs were not blinded to which scale they were administering. Oral administration of self-report tools involves an interaction between the questionnaire, the respondent and the interviewer which can lead to potential biases [27]. These areas of potential bias were mitigated through the careful training and monitoring of the data collection method used throughout the study. In the previous study conducted in the UK, eligible participants were invited to take part by the researcher and written informed consent was obtained. EPDS scores were measured online at about 21 weeks gestation.

Statistical analysis

All statistical analysis was run using R [28]. To compare the demographic and pregnancy characteristics independent two-sample t-tests were used. To investigate differences and similarities in the EPDS and the SRQ-20 within the Gambian sample, a Spearman's correlation

For peer review only - http://bmjopen.bmj.com/site/about/guidelines.xhtml

test was conducted, differences in the distributional properties were examined and the average score of the EPDS-3 subscale, and its contribution to the total EPDS score of the sample, was computed. Individual item frequencies on the SRQ-20 (# of yes responses out of total responses) were also calculated. To explore the impact of the somatic SRQ-20 items, the average score of the combined somatic items and their contribution to the total SRQ-20 score was calculated. Previous research has validated optimum cut-off scores for identifying perinatal CMDs within different populations in Africa [7,17]. The scores of 6, 7 and 8 were chosen as cut-offs for the SRQ-20, based on previous validation research in Ethiopia and Malawi [9,10]. Proportions of specific agreement and kappa statistics based on the identification of participants below or above these different cut offs were calculated based on methods explained by Cicchetti and Feinstein [29] and then Kappas were calculated.

To investigate differences across countries using the EPDS, differences in the distributional properties were explored. Three cut-off scores, 9, 10 and 12, were chosen for the EPDS based on previous research in the UK [6] and across Africa [17]. The average score of each item and of the EPDS-3 subscale was calculated. Its contribution to the total EPDS score of the sample was also computed. Differences between the total EPDS score between the UK and The Gambia were calculated using a Mann-Whitney U test.

Patient and Public Involvement

We engaged with patients and the public and relevant stakeholders in The Gambia to inform the research approach. This included how to best administer the questionnaires, the best way to recruit participants and potential cultural considerations which need to be made. To do this we held a half day meeting in the Gambia with delegates from the Ministry of Health and Social Welfare, the

For peer review only

For peer review only - http://bmjopen.bmj.com/site/about/guidelines.xhtml

National Centre for Arts and Culture, a local obstetrician, patient advocate groups and students in Public Health and Psychiatric Nursing. In general, all stakeholders felt this was a worthy and important endeavour as maternal mental health is a relatively unexplored area in The Gambia. One crucial point which was discussed was how we would sensitively manage and appropriately sign post women who report any concerning symptoms, such as suicidal ideation or domestic violence. It was decided that if this were to happen, the woman would receive in the moment front line counselling to talk through these issues with the RAs who are both trained psychiatric nurses. Then, if needed, she would be referred on to the Community Mental Health Team for further management. For women that report domestic violence emergencies and cases that require immediate intervention, the RAs would connect the woman with the Gender Based Violence focal person. For other cases, the RAs would refer the woman to the One Stop Center at Serekunda General Hospital or Edward Francis Small Teaching Hospital.

RESULTS

Demographic Information

Table 1 shows the demographic characteristics of the Gambian and UK samples. UK participants were significantly older ($p < 0.001$, 95%CIs [5.4, 7.2], $d = 1.4$) and had fewer children ($p < 0.001$, 95%CIs [0.6, 1.2], $d = 0.6$) and pregnancies ($p < 0.001$, 95%CIs [2.0, 3.6], $d = 0.9$) than the Gambian participants.

Table 1. Demographic Information for Gambian and UK Samples

	**Gambian Sample (n=221)**	**UK Sample (n=314) *M (SD)***

For peer review only - http://bmjopen.bmj.com/site/about/guidelines.xhtml

	M (SD)	
**Age***	27.3 (5.9)	33.4 (4.3)
**GA**	22.9 (5.5)	21.1 (1.4)
**Parity***	2.3 (2.1)	1.5 (0.7)
**Gravida***	3.5 (2.25)	2.0 (2.0)

**Gambian Sample**	**Total** *n (% of 221)*
**Marital Status**	
Single/Divorced/Widowed	6 (3%)
Married (Monogamous)	160 (72%)
Married (Polygamous)	55 (25%)
**Education Level**	
None	13(6%)
Informal (Arabic)	101 (46%)
Primary	29 (13%)
Secondary/Tertiary	78 (35%)
**Occupation**	
Housewife	138 (62%)
Other	82 (37%)
**Husband's Occupation**	
Skilled work	97(44%)
Manual/Trade Work	122 (55%)

**UK Sample**	***Total sample of 368****
**Marital Status**	***n(% out of 314)***
Married/Civil Partnership	233 (74%)
Living with Partner	64 (20%)
Single	11 (4%)
Other	6 (2%)
**Education Level**	***n(% out of 312)***
Left before GCSE/O-Levels	2 (1%)
GCSE/O-Levels	16 (5%)
A-Levels	23 (7%)
Vocational Training	13 (4%)
University Degree	159 (51%)
Higher Degree	102 (32%)
**Occupation**	***n(% out of 314)***
Professional	197 (63%)
Managerial	46 (15%)
Skilled Non-Manual	16 (5%)

For peer review only

Skilled Manual	13 (4%)
Partly Skilled	10 (3%)
Not Applicable	22 (7%)
Other	10 (3%)
**Ethnic Group**	***n(% out of 314)***
Caucasian	209 (67%)
Indian/Pakistani/Bangladeshi	21 (7%)
Middle Eastern	12 (4%)
Afro/Afro-Caribbean	12 (4%)
South American/Hispanic	4 (1%)
Far Eastern	3 (1%)
Mixed	12 (4%)
Other	41 (13%)

Note. GA = Gestational Age. Primip = Primiparious. Housewife includes women who stated they did not have a job ("None"). All categories were informed by the categorizations used by the Ministry of Health and Social Welfare (MoHSW) in The Gambia. GCSE = General Certificate of Secondary Education. UK participants with missing demographic data (n = 54) were taken out for this summary. *Indicates a significant difference ($p<0.05$) between the groups using independent samples t-tests. **The total sample in the previously conducted study was 368 but there was missing demographic data (54 participants). The total used for each category is included in the table.

Comparing the EPDS and the SRQ-20 in The Gambian Sample

The overall total scores for both scales were significantly moderately correlated $r_s = 0.6$, $p < 0.001$ (See Supplementary Material 3). Figure 1 compares the distributions of the total EPDS and SRQ-20 scores and the proportion of women above different cut-off scores.

Figure 1. Histogram of different cut-off scores for EPDS and SRQ-20.

There was also low agreement between the two scales in the Gambian sample in identifying those above the various cut-offs scores chosen (kappa's ranging from 0.05-0.25). The average positive agreement – when both scales detected a participant above the cut-off – was low (28%). Negative agreement – when both identified the participant as below the cut-off – was higher (67%).

For peer review only - http://bmjopen.bmj.com/site/about/guidelines.xhtml

The average overall agreement – positive and negative agreement combined – was 54%. Supplementary Material 4 displays the proportion of participants identified using different cut-off scores, proportions of specific agreement and kappa's between the SRQ-20 and the EPDS.

Table 2 shows the responses of the Gambian sample to the individual items on the SRQ-20. The somatic symptom items had a higher average score (*M* = 4.0, *SD* = 2.0) than psychological/cognitive/functional symptom items (*M* = 3.0, *SD* = 2.5). The somatic symptom items contributed 63% to the total SRQ-20 score. The psychological/cognitive/functional symptom items contributed 37%.

Table 2. SRQ-20 Item, Item Category Frequency

SRQ20 Item		Yes *n* (%)
1	Do you often have headaches?	167 (76%)
2	Is your appetite poor?	121 (55%)
3	Do you sleep badly?	93 (42%)
4	Are you easily frightened?	82 (37%)
5	Do your hands shake?	24 (11%)
6	Do you feel nervous, tense or worried?	89 (40%)
7	Is your digestion poor?	78 (35%)
8	Do you have trouble thinking clearly?	58 (26%)
9	Do you feel unhappy?	94 (43%)
10	Do you cry more than usual?	20 (9%)
11	Do you find it difficult to enjoy your daily activities?	45 (20%)
12	Do you find it difficult to make decisions?	74 (33%)
13	Is your daily work suffering?	80 (36%)
14	Are you unable to play a useful part in life?	60 (27%)
15	Have you lost interest in things?	74 (33%)
16	Do you feel that you are a worthless person?	37 (17%)
17	Has the thought of ending your life been on your mind?	10 (5%)
18	Do you feel tired all the time?	116 (52%)
19	Do you have uncomfortable feelings in your stomach?	121 (55%)
20	Are you easily tired?	109 (49%)
**Item Category**		***M (SD)***
1–3, 5, 7, 18–20	*Somatic Symptoms	4.0 (2.0)

For peer review only - http://bmjopen.bmj.com/site/about/guidelines.xhtml

4, 6, 8, 9–17	*Psychological/cognitive/functional Symptoms	3.0 (2.5)

Note. *Categorisation of items based on (Stewart et al., 2009[10])

Comparing EPDS scores in The Gambia and the UK

The UK average EPDS score (M = 6.5, SD = 4.4, 95%CI[6.1, 6.9]) was significantly higher than the Gambian average score (M = 4.4, SD = 3.7, 95% CI[3.9, 4.9] (p < 0.001, 95%CIs [-3.0, -1.0], *Cliff's delta* = -0.3) (p < 0.001 95%CIs [1.5, 2.8], d = 0.5). A Mann-Whitney U Test was used because the UK scores were slightly positively skewed (skewness = 0.63), while the Gambian EPDS scores were highly positively skewed (skewness = 1.37), reflecting a higher frequency of lower scores in the Gambian sample. Figure 2 shows the distribution of total EPDS scores in the Gambian and UK samples as well as the proportion above the chosen cut-off scores.

Figure 2. EPDS distributions and cut-off score for UK and Gambia samples.

13% of UK participant's EPDS scores were ≥12 compared to 5% in the Gambian sample. With a cut-off of ≥ 10, 27% of UK versus 10% of Gambian participants were identified and when using the cut-off of ≥9, 34% UK versus 13% Gambian participants were identified (Figure 2). Thus, with each cut-off, two to three times more women scored above the selected cut-off score from the UK sample compared with the Gambian sample.

Table 3 shows how the two samples scored on the individual items on the EPDS. In the Gambian sample the item scores were generally lower. The Gambian sample had a significantly higher average score (M = 0.4, SD = 0.6) compared to the UK sample (M = 0.3, SD = 0.5) only on the reverse coded item 2 (I have looked forward with enjoyment to things), (p < 0.004 95%CIs [0.3, 0.1], d = 0.3). For all other significant differences, the UK sample had a higher item average

For peer review only - http://bmjopen.bmj.com/site/about/guidelines.xhtml

(See Table 3). The EPDS includes an anxiety subscale (EPDS-3) consisting of items 3, 4 and 5 (Kabir et al. 2008; Mitchell 2009). This subscale average was also significantly higher for the UK sample (M = 3.5, SD = 2.0) compared with the Gambia sample (M = 1.7, SD = 1.7) ($p < 0.004$, 95%CIs [1.5, 2.1], d = 0.9). The anxiety subscale scores contributed 54% to the total UK EPDS score. For the Gambian sample the subscale contributed to 39% of the total EPDS score. Two of the most endorsed items in The Gambian sample were within the anxiety subscale (items 4 and 5).

Table 3. EPDS Item and Subscale Averages for the UK and Gambia Samples

		**UK Sample**	**Gambia Sample**
Item #		***M*(SD)**	***M*(SD)**
1	I have been able to laugh and see the funny side of things	0.2 (0.5)	0.3 (0.5)
2	I have looked forward with enjoyment to things*	0.3 (0.5)	0.4 (0.6)
3	I have blamed myself unnecessarily when things went wrong*	1.2 (0.8)	0.4 (0.7)
4	I have been anxious or worried for no good reason*	1.4 (0.9)	0.7 (0.8)
5	I have felt scared or panicky for no good reason*	0.9 (0.8)	0.6 (0.9)
6	Things have been getting on top of me*	1.0 (0.8)	0.6 (0.8)
7	I have been so unhappy that I have had difficulty sleeping	0.4 (0.7)	0.5 (0.8)
8	I have felt sad or miserable*	0.6 (0.8)	0.5 (0.7)
9	I have been so unhappy that I have been crying	0.5 (0.6)	0.3 (0.7)
10	The thought of harming myself had occurred to me	0.1 (0.3)	0.1 (0.3)
	**Anxiety subscale (3,4,5)***	3.5 (2.0)	1.7 (1.7)
	**All other items**	2.7 (2.6)	3.0 (2.9)
	**Total Score***	**6.5 (4.4)**	**4.4 (3.7)**

Note. Bonferroni adjusted alpha level of .004 was used per test (.05/13). **indicates a significant difference when using an independent sample t-test at p < 0.004*

DISCUSSION

For peer review only - http://bmjopen.bmj.com/site/about/guidelines.xhtml

When comparing scores on the EPDS between the Gambian and UK samples, the UK participants had significantly higher total EPDS scores and 13% of UK participant's scores were met threshold for high levels of symptoms (≥12) compared with only 5% of the Gambian sample. This could, of course, be taken at face value to indicate potentially higher levels of CMS symptoms in the UK population. However, the comparison of EPDS and SRQ-20 scores reveals an alternative view. While both scales were significantly moderately correlated ($r_s = 0.6$, $p < 0.00$), they had different distributions and only 54% overall agreement. Additionally, when using previously validated cut-off scores from other contexts, only 5% of Gambian participants met threshold for high levels of symptoms with the EPDS, while that number rose to 42% when the SRQ-20 was used.

The aim of this comparative cut-off score analysis was to investigate how various cut-offs scores validated in other perinatal contexts would compare in our sample. As has been shown in similar work around the world the two scales generally perform quite differently within the same context and the same scale performs quite differently across two different contexts. It may be that the EPDS significantly underestimates the incidence of CMD symptoms in this Gambian sample due to how it is administered, the response format and cultural differences in understandings a presentation of perinatal CMD symptoms as has been found in other similar perinatal populations (e.g.,[30,31]). It may also be that the EPDS does not capture the somatic way in which CMD symptoms are experienced. However, without a clinical interview to compare against, it is difficult to know the reason for the observed differences.

Somatic symptoms, rather than those of depressive mood, were more frequently endorsed by our Gambian sample. This may be in part because of differences of language. Different ways of describing the world can lead speakers of different languages to have different ways of thinking

For peer review only - http://bmjopen.bmj.com/site/about/guidelines.xhtml

about the world [32]. For example, Item 2 (I have looked forward with enjoyment to things) was the only item where the average score of the Gambian sample was significantly higher than the average score of the UK sample. It may be that the idea of anticipating or expecting enjoyment in your future might go against ideas of humility and patience, and faith in God (any plans for the future are normally concluded with 'inshallah', God willing).

Items related to sadness or crying were some of the least endorsed by the Gambian participants. Differences in CMD symptom presentation when measured using translated self-report measures may not reflect Gambian indigenous understandings of symptom causation and, in fact, these indigenous understandings may contrast with biomedical perspectives [33]. For example, a systematic review found that local language versions of the EPDS in LMICs had lower precision for identifying true cases of perinatal CMDs compared to the original English version [30]. Additionally, Molenaar et al. [34] found that in a perinatal population in rural Ethiopia, most women recognised the existence of perinatal mental distress states, but did not call such distress a discrete illness, such as depression. Instead, these mental distress states were generally seen as non-pathological reactions to difficult circumstances.

Some researchers have raised the concern that measuring somatic CMD symptoms in populations with high levels of physical disease might mistakenly attribute these symptoms to a depressive syndrome [15]. In a perinatal population this may present an even more difficult problem as somatic symptoms related to pregnancy are expected to be experienced and change throughout this time. However, an important study by Stewart et al. [10], found that the inclusion of somatic items did not affect the test performance of the SRQ-20 in their Malawi postnatal population as validated by a semi-structured diagnostic interview for depressive disorder. This current study is in agreement with previous research that used the SRQ-20 in other perinatal

For peer review only - http://bmjopen.bmj.com/site/about/guidelines.xhtml

African populations and found the somatic items from the SRQ-20 were, indeed, the most frequently endorsed [9,10].

Previous research has also found that somatization of mental health disorders in general, and perinatal disorders in particular, is common in many African populations [5,9,25,35]. This supports the idea that in many non-western cultures the symptoms of dysphoric disorders seem to be mostly somatic and different from the Western systems [35]. Taken together, as has been found in previous studies [3,36,37], our results further emphasise how methods and understanding around measuring perinatal mental health symptoms developed in Western countries need to be employed with care when working within and across different cultures.

Limitations

While careful consideration and translation of the SRQ-20 and EPDS helped to ensure semantic validity of the scales for use in The Gambia, the scales have not been validated against a clinical gold-standard (clinical interview) to screen for possible CMD, highlighting a limitation of this work. Other studies conducted in sub-Saharan African countries with perinatal populations studies have shown good criterion validity of the SRQ-20 and the EPDS against the clinical interview [10,25,38–40]. However, validation against a clinical interview was difficult in The Gambia partly because there are few trained professionals (only two trained psychiatrists for the whole of the country) able to conduct these interviews in the two local languages. This limitation highlights an important consideration needed when conducting validation studies in low resource settings with few local trained health professionals Even though the scales were not validated we feel the comparison of the scales' performances when using possible cut-off scores is an interesting and helpful exploratory process.

For peer review only - http://bmjopen.bmj.com/site/about/guidelines.xhtml

For the purposes of this comparative analysis, we combined the scores from the Mandinka and Wolof languages. Mandinka is a Mande language and Wolof is classified as a Niger-Congo language and there is a mutual influence between them. If validation were to be done in the future, ideally each of the languages would be assessed separately.

How the EPDS was administered was different in the two settings. The EPDS was self-completed in written form in the UK versus delivered and responded to orally in The Gambia due to the low literacy rate in the population. Other factors include differences in the recruitment process, purpose, and setting (hospital versus community), and in demographic factors such as age and parity. These differences were partly because existing data from a previously conducted study in England was used. Therefore, these other factors might explain, in part, some of the differences reported on the EPDS between participants in The Gambia versus the UK. However, it seems unlikely that these factors alone could explain the major differences in response.

The SRQ-20's binomial response format, compared to the EPDS likert-scale response format, may have influenced the differences observed in the performance of these two tools. The binomial response format is more easily administered and understood by respondents with low literacy [37]. For example, a study comparing these two scales in a Ghanaian pregnant population found that it was important to alter the format of the EPDS in Ghana, for it to be understandable to women [41]. Responses like 'hardly ever' and 'sometimes' might have been difficult to distinguish when administered orally.

CONCLUSION

In conclusion, these findings support studies conducted with perinatal populations in other parts of Africa [9,10,25,42]. This study is able to add to this growing body of work by exploring

For peer review only - http://bmjopen.bmj.com/site/about/guidelines.xhtml

differences within a new cultural context. While caution should be taken when generalising these results, this study is one of the first to explore the expression of antenatal mental health symptoms in The Gambia when using two common CMD measurement tools. It will be of interest in the future to explore further the cultural reasons for the discrepancies found.

Crucially, this study helps to further underline the importance of investigating differences in scale performance within and across cultures to ensure that the measurement scale chosen is sensitive to cultural differences in the understanding and presentation of perinatal common mental disorders. Without careful exploration of these differences, research in this area may have detrimental consequences in detection and therefore treatment of perinatal mental health in LMICs.

Funding

This study was funded by the MRC-AHRC Global Public Health: Partnership Awards scheme (MR/R024618/1) awarded to Professor Lauren Stewart. This writing of this manuscript was supported by a South East Network for Social Sciences/The Economic and Social Research Council funded postdoctoral fellowship awarded to Dr Katie Rose Sanfilippo (Grant Reference Number: ES/V010158/1).

Competing interests

The authors declare no competing interests

Author Contributions

The manuscript was prepared by KRMS and all authors approved the final draft. KRMS and BM helped with data management and data collection materials. BD provided local knowledge,

For peer review only - http://bmjopen.bmj.com/site/about/guidelines.xhtml

expertise in health provision and healthcare in The Gambia, and organisation in the field liaising with antenatal clinics. HC provided management of the funds and transportation in The Gambia. HBH helped complete all data collection and input and provided knowledge of mental health and reproductive health in the Gambia. KRMS completed data cleaning and analysis. VC provided methodological and statistical expertise. VG and PR provided expertise in perinatal mental health. IC provided expertise on culture and language and BM, HC and BD provided expertise on Gambian culture and language. RAC was responsible for all data collection and management for the UK sample. All authors, except VG, RAC and IC, spent time in The Gambia supervising the project on the ground. LS is the grant holder and principal investigator and oversaw all aspects of the study.

Data sharing statement

De-identified participant data that correspond to the results reported in this article are available upon reasonable request from the corresponding author (katie-rose.sanfilippo@city.ac.uk).

Ethical approval and consent to participate

All procedures performed in studies involving human participants were in accordance with the ethical standards of the institutional and/or national research committee and with the 1964 Helsinki Declaration and its later amendments or comparable ethical standards. All procedures involving human participants were approved by the Goldsmiths University Ethics Committee (1392), The Gambia Government/MRC Gambia joint Ethics Committee (R018 015V2) and the Australian National University Ethics Committee (2018/235). Members of the research team carried out the

For peer review only
For peer review only - http://bmjopen.bmj.com/site/about/guidelines.xhtml

consenting and conduct of this study orally. It was emphasised that any participant was able to withdraw from the study at any point without any consequences.

Acknowledgements

We would like to thank the women and clinicians in the antenatal clinics in the Gambia and at Queen Charlotte's and Chelsea Hospital in the UK. Thank you also to Pa Bakary Sonko and Charlotte Hanlon for their advice and support, Giles Partington for help with analysis advice and Jane Offerman for helping with administration.

REFERENCES

1 Glover V, O'Donnell KJ, O'Connor TG, *et al.* Prenatal maternal stress, fetal programming, and mechanisms underlying later psychopathology - A global perspective. *Dev Psychopathol* 2018;**30**:843–54. doi:10.1017/S095457941800038X

2 Fisher J, de Mello MC, Patel V, *et al.* Prevalence and determinants of common perinatal mental disorders in women in low- and lower-middle-income countries: A systematic review. *Bull World Health Organ* 2012;**90**:139–49. doi:10.2471/BLT.11.091850

3 Kirmayer LJ. Cultural Variations in the Clinical Presentation of Depression and Anxiety: Implications for Diagnosis and Treatment. *J Clin Psychiatry* 2001;**62**:22–8.

4 Oates MR, Cox JL, Neema S, *et al.* Postnatal depression across countries and cultures: A qualitative study. *British Journal of Psychiatry* 2004;**184**:s10–6.

5 Senturk V, Hanlon C, Medhin G, *et al.* Impact of perinatal somatic and common mental disorder symptoms on functioning in Ethiopian women: The P-MaMiE population-based cohort study. *J Affect Disord* 2012;**136**:340–9. doi:10.1016/j.jad.2011.11.028

6 Cox JL, Holden J, Henshaw C. *Perinatal mental health: The Edinburgh Postnatal Depression Scale (EPDS) manual*. 2nd ed. London, UK: : The Royal College of Psyciatrists Publications 2014.

7 Ali GC, Ryan G, De Silva MJ. Validated screening tools for common mental disorders in low and middle income countries: A systematic review. *PLoS One* 2016;**11**:e0156939. doi:10.1371/journal.pone.0156939

8 Beusenberg M, Orley J. A user's guide to the Self Reporting Questionnaire (SRQ). *World Health Organization, Geneva* 1994;:1–84.http://apps.who.int/iris/bitstream/handle/10665/61113/?sequence=1

For peer review only - http://bmjopen.bmj.com/site/about/guidelines.xhtml

9 Hanlon C, Medhin G, Alem A, *et al.* Detecting perinatal common mental disorders in Ethiopia: Validation of the self-reporting questionnaire and Edinburgh Postnatal Depression Scale. *J Affect Disord* 2008;**108**:251–62. doi:10.1016/j.jad.2007.10.023
10 Stewart RC, Kauye F, Umar E, *et al.* Validation of a Chichewa version of the Self-Reporting Questionnaire (SRQ) as a brief screening measure for maternal depressive disorder in Malawi, Africa. *J Affect Disord* 2009;**112**:126–34. doi:10.1016/j.jad.2008.04.001
11 Okulate GT, Olayinka MO, Jones OBE. Somatic symptoms in depression: Evaluation of their diagnostic weight in an African setting. *British Journal of Psychiatry* 2004;**184**:422–7. doi:10.1192/bjp.184.5.422
12 Gelaye B, Rondon MB, Araya R, *et al.* Epidemiology of maternal depression, risk factors, and child outcomes in low-income and middle-income countries. *Lancet Psychiatry* 2016;**3**:973–82. doi:10.1016/S2215-0366(16)30284-X
13 Hulley SB, Cummings SR, Browner WS, *et al. Designing clinical research: an epidemiologic approach*. 2nd ed. Philadelphia: : Lippincott Williams & Wilkins 2001.
14 Kelsey JL, Whittemore AS, Evans AS, *et al. Methods in observational epidemiology*. Oxford: : Oxford University Press 1996.
15 Cox JL, Holden JM, Sagovsky R. Detection of postnatal depression: Development of the 10-item Edinburgh Postnatal Depression Scale. *The British Journal of Psychiatry* 1987;**150**:782–6. doi:10.1192/bjp.150.6.782
16 Kozinszky Z, Dudas RB. Validation studies of the Edinburgh Postnatal Depression Scale for the antenatal period. *J Affect Disord* 2015;**176**:95–105. doi:10.1016/j.jad.2015.01.044
17 Tsai AC, Scott JA, Hung KJ, *et al.* Reliability and validity of instruments for assessing perinatal depression in African settings: Systematic review and meta-analysis. *PLoS One* 2013;**8**:e82521. doi:10.1371/journal.pone.0082521
18 Coleman R, Morison L, Paine K, *et al.* Women's reproductive health and depression: A community survey in The Gambia, West Africa. *Soc Psychiatry Psychiatr Epidemiol* 2006;**41**:720–7. doi:10.1007/s00127-006-0085-8
19 Bartram C. *Early infant social behaviour and maternal perinatal depression in Kiang West , The Gambia*. 2018.
20 Nabwera HM, Moore SE, Mwangome MK, *et al.* The influence of maternal psychosocial circumstances and physical environment on the risk of severe wasting in rural Gambian infants: A mixed methods approach. *BMC Public Health* 2018;**18**:109. doi:10.1186/s12889-017-4984-2
21 Sanfilippo KRM, McConnell B, Cornelius V, *et al.* Community psychosocial music intervention (CHIME) to reduce antenatal common mental disorder symptoms in The Gambia: a feasibility trial. *BMJ Open* 2020;**10**:e040287. doi:10.1136/bmjopen-2020-040287
22 Mitchell AJ. The 3 item anxiety subscale of the Edinburgh Postpartum Depression Scale may detect postnatal depression as well as the 10 item full scale. *Evid Based Ment Health* 2009;**12**:44. doi:10.1136/ebmh.12.2.44

For peer review only - http://bmjopen.bmj.com/site/about/guidelines.xhtml

23 Sawyer A, Ayers S, Smith H, *et al.* Women's experiences of pregnancy, childbirth, and the postnatal period in the Gambia: A qualitative study. *Br J Health Psychol* 2011;**16**:528–41. doi:10.1348/135910710X528710

24 World Health Organization. Process of translation and adaptation of instruments. 2014.

25 Hanlon C, Medhin G, Alem A, *et al.* Measuring common mental disorders in women in Ethiopia. *Soc Psychiatry Psychiatr Epidemiol* 2008;**43**:653–9. doi:10.1007/s00127-008-0342-0

26 The Gambia Bureau of Statistics (GBOS) and ICF International. The Gambia Demographic and Health Survey 2013. Banjul, The Gambia and Rockville, Maryland, USA: 2014.

27 Bowling A. Mode of questionnaire administration can have serious effects on data quality. *J Public Health (Bangkok)* 2005;**27**:281–91. doi:10.1093/pubmed/fdi031

28 R Core Team. R: A language and environment for statistical computing. 2013.http://www.r-project.org/

29 Cicchetti D V., Feinstein AR. High agreement but low kappa: II. Resolving the paradoxes. *J Clin Epidemiol* 1990;**43**:551–8. doi:10.1016/0895-4356(90)90159-M

30 Shrestha SD, Pradhan R, Tran TD, *et al.* Reliability and validity of the Edinburgh Postnatal Depression Scale (EPDS) for detecting perinatal common mental disorders (PCMDs) among women in low-and lower-middle-income countries: A systematic review. *BMC Pregnancy Childbirth* 2016;**16**:1–19. doi:10.1186/S12884-016-0859-2/FIGURES/3

31 Bluett-Duncan M. *A cross-cultural comparison of the role of maternal mental health in the prediction of infant cognitive development and empirical investigation of the role of early caregiving in India.* 2021. doi:10.17638/03129982

32 Boroditsky L. Linguistic Relativity. In: Nadel L, ed. *Encyclopedia of Cognitive Science*. Chichester: : John Wiley & Sons, Ltd 2006. doi:10.1002/0470018860.s00567

33 McConnell BB. *Music, health, and power: Singing the unsayable in The Gambia*. New York, NY: : Routledge 2020. doi:10.4324/9780367312732

34 Molenaar J, Hanlon C, Alem A, *et al.* Perinatal mental distress in a rural Ethiopian community: A critical examination of psychiatric labels. *BMC Psychiatry* 2020;**20**. doi:10.1186/s12888-020-02646-5

35 Halbreich U, Karkun S. Cross-cultural and social diversity of prevalence of postpartum depression and depressive symptoms. *J Affect Disord* 2006;**91**:97–111. doi:10.1016/j.jad.2005.12.051

36 Kortmann F. Problems in communication in transcultural psychiatry: The self reporting questionnaire in Ethiopia. *Acta Psychiatr Scand* 1987;**75**:563–70. doi:10.1111/j.1600-0447.1987.tb02836.x

37 Sweetland AC, Belkin GS, Verdeli H. Measuring depression and anxiety in sub-Saharan Africa. *Depress Anxiety* 2014;**31**:223–32. doi:10.1002/da.22142

38 Tesfaye M, Hanlon C, Wondimagegn D, *et al.* Detecting postnatal common mental disorders in Addis Ababa, Ethiopia: Validation of the Edinburgh Postnatal Depression Scale and Kessler Scales. *J Affect Disord* 2010;**122**:102–8. doi:10.1016/j.jad.2009.06.020

For peer review only - http://bmjopen.bmj.com/site/about/guidelines.xhtml

39 van der Westhuizen C, Wyatt G, Williams JK, *et al.* Validation of the Self Reporting Questionnaire 20-Item (SRQ-20) for use in a Low- and Middle-Income country emergency centre setting. *Int J Ment Health Addict* 2016;**14**:37–48. doi:10.1007/s11469-015-9566-x

40 van der Westhuizen C, Brittain K, Koen N, *et al.* Sensitivity and specificity of the SRQ-20 and the EPDS in diagnosing major depression ante- and postnatally in a South African birth cohort study. *Int J Ment Health Addict* 2018;**16**:175–86. doi:10.1007/s11469-017-9854-8

41 Weobong B, Akpalu B, Doku V, *et al.* The comparative validity of screening scales for postnatal common mental disorder in Kintampo, Ghana. *J Affect Disord* 2009;**113**:109–17. doi:10.1016/j.jad.2008.05.009

42 Chorwe-Sungania G, Chipps J. Validity and utility of instruments for screening of depression in women attending antenatal clinics in Blantyre district in Malawi. *South African Family Practice* 2018;**60**:55. doi:10.4102/safp.v60i4.4888

For peer review only

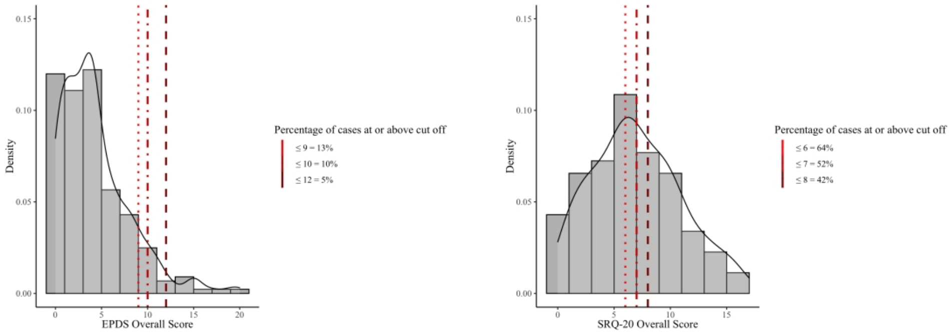

Figure 1. Histogram of different cut-off scores for EPDS and SRQ-20.

1587x529mm (72 x 72 DPI)

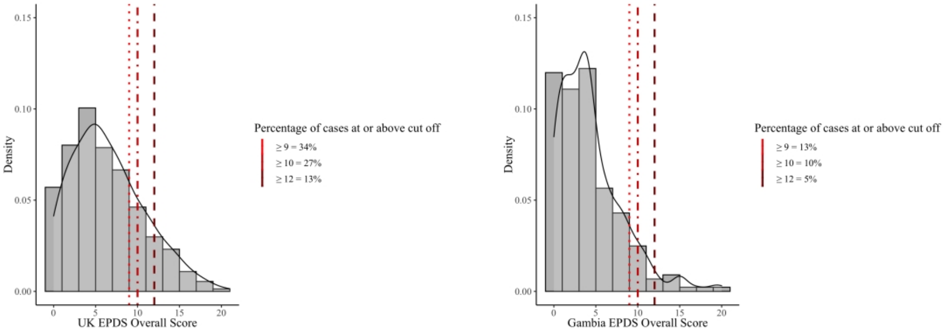

Figure 2. EPDS distributions and cut-off score for UK and Gambia samples.

1587x529mm (72 x 72 DPI)

Supplementary Material 1

STROBE Statement—Checklist of items that should be included in reports of ***cross-sectional studies***

	Item No	Recommendation	Page Number
**Title and abstract**	1	(*a*) Indicate the study's design with a commonly used term in the title or the abstract	Pg 1
		(*b*) Provide in the abstract an informative and balanced summary of what was done and what was found	Pg 2
**Introduction**			
Background/rationale	2	Explain the scientific background and rationale for the investigation being reported	Pg 3
Objectives	3	State specific objectives, including any prespecified hypotheses	Pg 5
**Methods**			
Study design	4	Present key elements of study design early in the paper	Pg 5
Setting	5	Describe the setting, locations, and relevant dates, including periods of recruitment, exposure, follow-up, and data collection	Pg 5-6
Participants	6	(*a*) Give the eligibility criteria, and the sources and methods of selection of participants	Pg 5-6
Variables	7	Clearly define all outcomes, exposures, predictors, potential confounders, and effect modifiers. Give diagnostic criteria, if applicable	Pg 7-8
Data sources/ measurement	8*	For each variable of interest, give sources of data and details of methods of assessment (measurement). Describe comparability of assessment methods if there is more than one group	Pg 7-8
Bias	9	Describe any efforts to address potential sources of bias	Pg 8
Study size	10	Explain how the study size was arrived at	Pg 5-6
Quantitative variables	11	Explain how quantitative variables were handled in the analyses. If applicable, describe which groupings were chosen and why	Pg 8-9
Statistical methods	12	(*a*) Describe all statistical methods, including those used to control for confounding	Pg 8-9
		(*b*) Describe any methods used to examine subgroups and interactions	NA
		(*c*) Explain how missing data were addressed	Pg 8-9, Table 1
		(*d*) If applicable, describe analytical methods taking account of sampling strategy	NA
		(*e*) Describe any sensitivity analyses	NA
**Results**			
Participants	13*	(a) Report numbers of individuals at each stage of study—eg numbers potentially eligible, examined for eligibility, confirmed eligible, included in the study, completing follow-up, and analysed	Pg 10-11
		(b) Give reasons for non-participation at each stage	NA
		(c) Consider use of a flow diagram	NA
Descriptive data	14*	(a) Give characteristics of study participants (eg demographic, clinical, social) and information on exposures and potential confounders	Pg 10-11
		(b) Indicate number of participants with missing data for each	Pg 10-11

For peer review only - http://bmjopen.bmj.com/site/about/guidelines.xhtml
For peer review only

		variable of interest	
Outcome data	15*	Report numbers of outcome events or summary measures	Pg 10-15
Main results	16	(*a*) Give unadjusted estimates and, if applicable, confounder-adjusted estimates and their precision (eg, 95% confidence interval). Make clear which confounders were adjusted for and why they were included	Pg 12-15
		(*b*) Report category boundaries when continuous variables were categorized	Pg 12-15
		(*c*) If relevant, consider translating estimates of relative risk into absolute risk for a meaningful time period	NA
Other analyses	17	Report other analyses done—eg analyses of subgroups and interactions, and sensitivity analyses	Pg 12-15
**Discussion**			
Key results	18	Summarise key results with reference to study objectives	Pg 16
Limitations	19	Discuss limitations of the study, taking into account sources of potential bias or imprecision. Discuss both direction and magnitude of any potential bias	Pg 18-19
Interpretation	20	Give a cautious overall interpretation of results considering objectives, limitations, multiplicity of analyses, results from similar studies, and other relevant evidence	Pg 16- 19
Generalisability	21	Discuss the generalisability (external validity) of the study results	Pg 20
**Other information**			
Funding	22	Give the source of funding and the role of the funders for the present study and, if applicable, for the original study on which the present article is based	Pg 20

*Give information separately for exposed and unexposed groups.

Note: An Explanation and Elaboration article discusses each checklist item and gives methodological background and published examples of transparent reporting. The STROBE checklist is best used in conjunction with this article (freely available on the Web sites of PLoS Medicine at http://www.plosmedicine.org/, Annals of Internal Medicine at http://www.annals.org/, and Epidemiology at http://www.epidem.com/). Information on the STROBE Initiative is available at www.strobe-statement.org.

For peer review only

Supplementary Material 2

Table sup 2 Antenatal Clinic Names, Type, Area and Language

Antenatal Clinic	Clinic Type	Area Type	Language
Banjuliding Health Centre	Minor Health Clinic	Urban	Mandinka
Essau Distract Hospital	District hospital	Urban	Wolof
Fajikunda Health Centre	Major Health Clinic	Urban	Wolof
Gunjur Health Centre	Minor Health Clinic	Rural	Mandinka
Kafuta Health Centre	Minor Health Clinic	Rural	Mandinka
Kuntair Health Centre	Minor Health Clinic	Rural	Wolof
Pirang Health Centre	Minor Health Clinic	Rural	Mandinka
Serekunda Health Centre	Major Health Clinic	Urban	Wolof
Sinchu Baliya Health Post	Community Clinic/Health Post	Urban	Wolof
Sukuta Health Centre	Minor Health Clinic	Urban	Mandinka

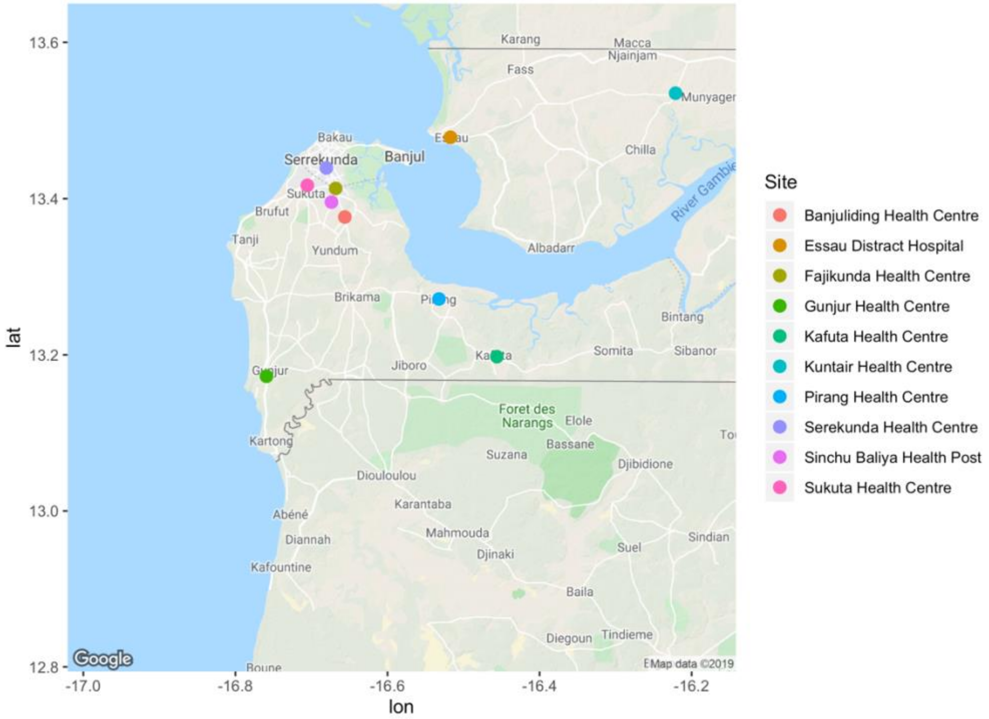

Fig sup 2. A map of the participating antenatal clinics described in Table sup 2.

Supplementary Material 3

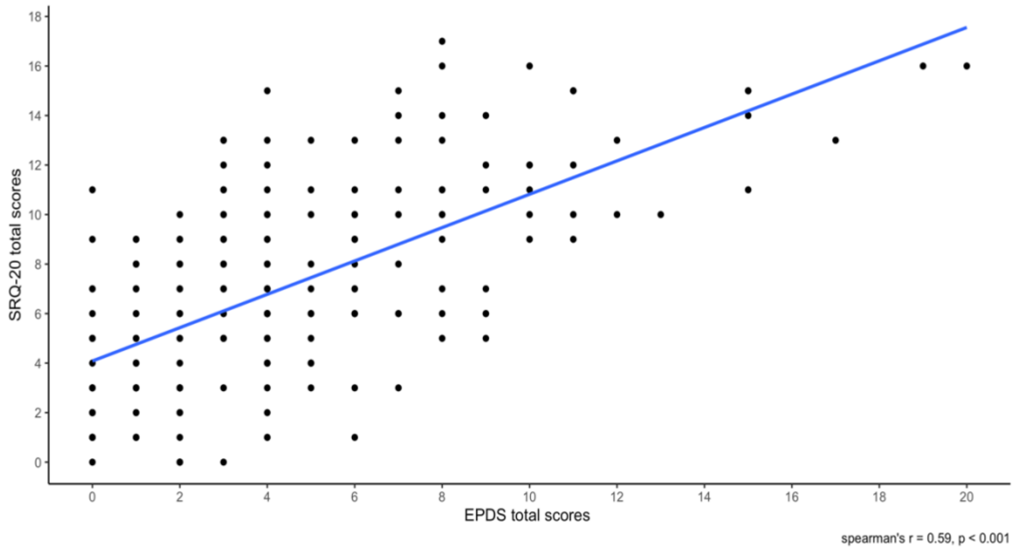

Fig Sup.3. Scatterplot of the correlation between Gambia EPDS and SRQ-20 total scores.

Supplementary Materials 4

Table Sup.4.

Proportions of Specific Agreement and Kappa's

**Cut-offs compared**	**Positive Agreement *n* (%)**	**Negative Agreement *n* (%)**	**Overall Agreement *n* (%)**	**Dis-agreement *n* (%)**	**Cohen's Kappa**
EPDS ≥ 12 & SRQ20 ≥ 8	10 (19)	128 (76)	138 (60)	83 (36)	0.12
EPDS ≥ 12 & SRQ20 ≥ 7	10 (16)	107 (67)	117 (51)	104 (45)	0.09
EPDS ≥ 12 & SRQ20 ≥ 6	10 (13)	80 (55)	90 (39)	131 (57)	0.05
EPDS ≥ 10 & SRQ20 ≥ 8	21 (37)	128 (78)	149 (65)	72 (31)	0.25
EPDS ≥ 10 & SRQ20 ≥ 7	21 (31)	107 (70)	128 (56)	93 (41)	0.18
EPDS ≥ 10 & SRQ20 ≥ 6	21 (26)	80 (57)	101 (44)	120 (52)	0.11
EPDS ≥ 9 & SRQ20 ≥ 8	24 (40)	124 (77)	148 (65)	73 (32)	0.25
EPDS ≥ 9 & SRQ20 ≥ 7	25 (35)	104 (69)	129 (56)	92 (40)	0.19
EPDS ≥ 9 & SRQ20 ≥ 6	27 (32)	79 (58)	106 (46)	115 (50)	0.14
**Average Agreement**	**28%**	**67%**	**54%**	**43%**	

Note. Percent positive agreement = 2(number both ≥ cut off)/2(number both ≥ cut off) + total number of disagreement; Percent negative agreement = 2(number both ≤ cut off)/2(number both ≤ cut off) + total number of disagreement (Cicchetti & Feinstein, 1990)

For peer review only
